# Benchmarking of Two Peptide Clean-Up Protocols: SP2 and Ethyl Acetate Extraction for Sodium Dodecyl Sulfate or Polyethylene Glycol Removal from Plant Samples before LC-MS/MS

**DOI:** 10.3390/ijms242417347

**Published:** 2023-12-11

**Authors:** Petra Martinková, Hana Konečná, Petr Gintar, Karolína Kryštofová, David Potěšil, Martin Trtílek, Zbyněk Zdráhal

**Affiliations:** 1 Mendel Centre for Plant Genomics and Proteomics, Central European Institute of Technology, Masaryk University, Kamenice 5, 625 00 Brno, Czech Republic; petra.martinkova@ceitec.muni.cz (P.M.); petr.gintar@ceitec.muni.cz (P.G.); karolina.krystofova@ceitec.muni.cz (K.K.); david.potesil@ceitec.muni.cz (D.P.); 2 National Centre for Biomolecular Research, Faculty of Science, Masaryk University, Kamenice 5, 625 00 Brno, Czech Republic; 3 Photon Systems Instruments, Spol. s r.o., Průmyslová 470, 664 24 Drásov, Czech Republic

**Keywords:** peptide clean-up, magnetic beads, SP2, ethyl acetate extraction, sodium dodecyl sulfate, detergent, polyethylene glycol, *Arabidopsis thaliana*, LC-MS/MS

## Abstract

The success of bottom-up proteomic analysis frequently depends on the efficient removal of contaminants from protein or peptide samples before LC-MS/MS. For a peptide clean-up workflow, single-pot solid-phase-enhanced peptide sample preparation on carboxylate-modified paramagnetic beads (termed SP2) was evaluated for sodium dodecyl sulfate or polyethylene glycol removal from *Arabidopsis thaliana* tryptic peptides. The robust and efficient 40-min SP2 protocol, tested for 10-ng, 250-ng, and 10-µg peptide samples, was proposed and benchmarked thoroughly against the ethyl acetate extraction protocol. The SP2 protocol on carboxylated magnetic beads proved to be the most robust approach, even for the simultaneous removal of massive sodium dodecyl sulfate (SDS) and polyethylene glycol (PEG) contaminations from AT peptide samples in respect of the LC-MS/MS data outperforming ethyl acetate extraction.

## 1. Introduction

Proteomic sample processing workflows can potentially introduce contaminating substances into protein or peptide solutions. Detergents or polymers might interfere with subsequent LC-MS/MS [1,2,3]. Their presence can lead to ion suppression, shifting the retention times or widening the chromatographic peaks, which can cause a decrease in the MS signal intensity. Such distorted chromatography can have a negative effect on peptide identification and quantification. Contaminants can originate from different steps of proteomics workflows. Depending on the source and nature of the impurities, different methods for the removal of contamination can be applied. For example, if the contamination originates from LC column bleeding or contaminated mobile phases, in-house-made salts for mobile phases or C18 purification cartridges can be used [4,5,6]. These cartridges can be suitable for the removal of the PEGs, however, SDS contamination would remain in the sample. In case of prominent keratin or tryptic peptide contamination, exclusion lists can be applied in order to not waste time on such peptides during the MS analysis [7]. Nevertheless, most frequently, contaminants originate directly from the sample or are introduced during the sample preparation procedure, and they should be removed prior to analysis.

Numerous methods for protein sample clean-up have been brought to attention. Well-established procedures based on protein precipitation, such as trichloroacetic acid/acetone or methanol–chloroform precipitation [8,9,10], are currently supplemented with solution-based techniques following the current trends directing to gel-free proteomic workflows, e.g., filter-aided sample preparation (FASP) [1] or the robust, fast, and low-cost SP3 protocol on paramagnetic carboxylate-modified beads [11]. However, based on different processing protocols (e.g., in-solution or on-beads digestion after immunoprecipitation or the incomplete performance of the above-mentioned techniques), hands-on peptide clean-up procedures should be available, too. More recently, magnetic-particle-based clean-up was implemented for contaminated peptides as well [12,13]. Similarly to SP3, the SP2 method is based on the immobilization of peptides onto the carboxylated paramagnetic beads [14]. This bond is formed between carboxyl groups on the beads and free amino groups on the molecules [15].

The most comprehensive study of the peptide clean-up protocol based on carboxylate-modified magnetic beads was published by Waas et al. [12]. The authors introduced the term SP2 for this workflow and systematically optimized and evaluated the key experimental parameters (e.g., the number of peptides and beads and working concentrations) for the best performance to remove detergents (Invitrosol, sodium lauryl sarcosinate, and sodium deoxycholate) and unidentified polymers. The binding capacity was markedly lower for peptides than for proteins; moreover, the particle-to-peptide ratio of 5:1 to 20:1 was recommended for the SP2. As far as the common digestion buffer removal is concerned, less than 1% of ammonium bicarbonate and approximately 20% of the original Tris concentration remained in the sample after SP2 clean-up. The workflow can also be applied to phosphopeptide and glycopeptide clean-up.

The method of Waas et al. [12] was slightly modified and published in Current Protocols [13]. The SP2 protocol was tested to remove many common contaminants from peptide samples, including salts, detergents (e.g., sodium dodecyl sulfate—SDS, Tween 20, and Triton), and polyethylene glycol (PEG). The key parameters for efficient peptide clean-up were the binding working volume of up to 50 μL and the particle-to-peptide ratio of 10:1 to 20:1. The Sera-Mag SpeedBeads with a second layer of magnetite performed equivalently regarding recovery and cleaning capacity as Sera-Mag Beads. Recently, the SP2 method utilizing carboxylate-modified magnetic beads was successfully applied to fractionate peptides by decreasing the isopropanol concentration. The method named CIF was published by Deng et al. [16].

A decade ago, Yeung and Stanley [17] published a rapid protocol for peptide clean-up, in which ethyl acetate extraction is used to remove detergents, such as SDS, octyl glucoside, Nonidet P-40, and Triton X-100, from protease digests. After aspirating the upper ethyl acetate layer containing the detergents, the cleaned peptides remain in the aqueous phase at the bottom of the tube.

Following common practice, all of the above-mentioned techniques were primarily tested on peptide samples originating from mammalian input material. However, plant samples represent a more complicated matrix, due to the presence of different classes of contaminants, which might interfere with all of the steps of the sample preparation procedure [18].

In this study, we evaluated two methods for peptide clean-up applied to the model plant *A. thaliana*. Tryptic peptides from whole-leaf lysates deprived of contaminations were supplemented with defined concentrations of SDS or PEG and processed in the following two workflows: the SP2 method using carboxylated paramagnetic beads or the ethyl acetate extraction method (SDS only). The SP2 protocol with unified conditions was evaluated to remove SDS or PEG at different peptide input levels covering four orders of magnitude. The outcomes provide a solid platform for broad applications of both peptide clean-up approaches, not only for plant samples. 

## 2. Results

Two alternative approaches, our SP2 protocol and ethyl acetate extraction (EE), were systematically benchmarked for the off-line clean-up of plant samples with regard to SDS. Additionally, the SP2 clean-up protocol was applied for the removal of PEG. The experiments were designed for peptide input amounts of 250 ng and 10 µg. Samples of *Arabidopsis thaliana* tryptic peptides, generated by FASP, were subsequently contaminated, as follows: (1) SDS to three levels of a final concentration of 0.1%, 1%, and 5%, or (2) PEG to three levels of a final concentration of 0.01%, 0.1%, and 1% (SP2 clean-up only). As an additional variable for the EE workflow, the optimal number of iterations for a given SDS concentration was also assessed for the clean-up of a 250-ng sample. The differences between the two clean-up methods were evaluated based on the LC-MS/MS data processed by the DIA-NN program using the following characteristics: (1) the number of quantified peptides, (2) the peptides’ signal characteristics (intensity, area, peak width, and retention time), and (3) the physical-chemical properties (hydrophobicity and molecular weight). The results were compared to the control peptide digest after FASP without any artificial contamination or clean-up. In the case of the 10-µg peptide input, the intensities were normalized according to the injection volume. Only the peptides quantified in at least two replicates of at least one sample category of the given comparison (control, clean-up of uncontaminated sample, or clean-up of samples with different levels of contamination) were used for the comparisons. The peptide intensity ratios were calculated to the control sample intensities, with the missing values being pairwise imputed by the minimum value observed for the given processing type to assess the qualitative changes.

The SP2 protocol was also tested for the clean-up of the 250-ng and 10-µg samples contaminated simultaneously by 5% SDS and 1% PEG. Finally, the SP2 protocol with no adjustments was applied for the clean-up of the 10-ng sample.

### 2.1. SDS Removal by Ethyl Acetate Extraction

In our common practice, three EE iterations are usually sufficient to clean up the samples from the residual SDS, e.g., after FASP, which might occur especially after processing higher amounts of protein loads. However, our aim was to evaluate the capabilities and limits of ethyl acetate extraction on different amounts of peptide samples; therefore, we tested this method with excessive SDS contaminations of up to 5% in combination with the increased number of iterations of up to 12.

**Figure 1:**
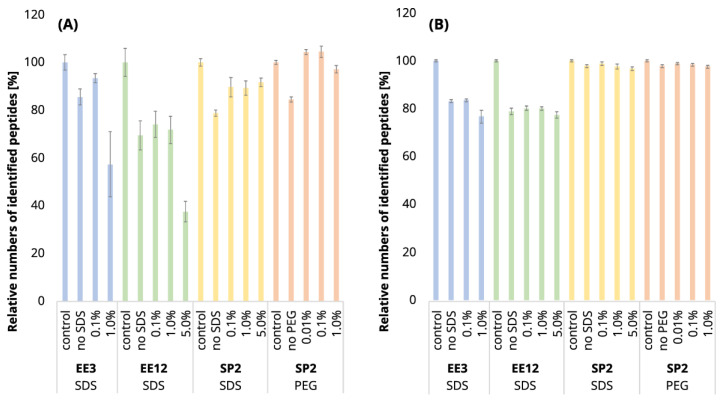
The mean numbers of identified peptides in 250-ng (**A**) and 10-µg (**B**) peptide inputs. The means were relatively compared to the control (100%). The standard deviations are displayed as error bars. Experiment description: EE3 or EE12 stands for 3 or 12 ethyl acetate extraction iterations, respectively; control means samples with no contamination and no EE or SP2 processing; no SDS/PEG means samples subjected to clean-up by either EE or SP2 processing with no added contamination; 0.01, 0.1%, 1%, or 5% represent samples with the given level of sodium dodecyl sulfate (SDS) or polyethylene glycol (PEG) contamination (*v*/*v*). See Table S1 for more details regarding the numbers of identified peptides.

**Figure 2:**
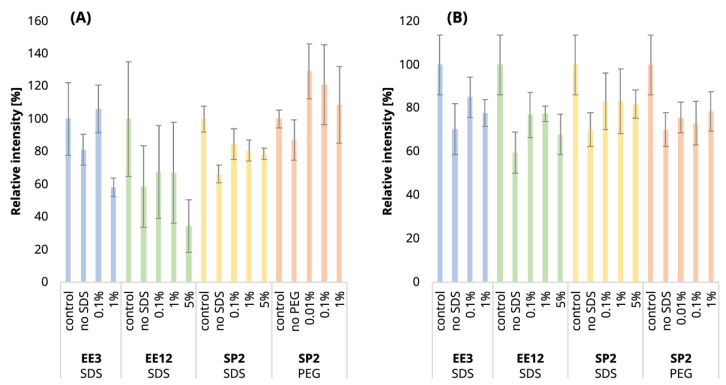
Mean intensity sums of quantified peptides in 250-ng (**A**) and 10-µg (**B**) peptide inputs. The mean sum intensities were relatively compared to the control (100%). The standard deviations are displayed as error bars. See Figure 1 for the experiments’ description.

It is expectable that the sample losses will increase with the higher number of EE iterations. Indeed, we observed a loss of 30.6% of the identified peptides after EE with 12 iterations of an uncontaminated 250-ng peptide input, while in the case of 3 EE iterations, it was only 14.5% (compared to the control sample, Figure 1A, blue and green bars; Table S1). The increased number of iterations was also reflected in the decrease in peptide intensities (Figure 2A, blue and green bars). After 3 iterations, the mean intensities sum decreased by 19%, but 12 EE iterations led to a reduction of 41%, on average.

**Figure 3:**
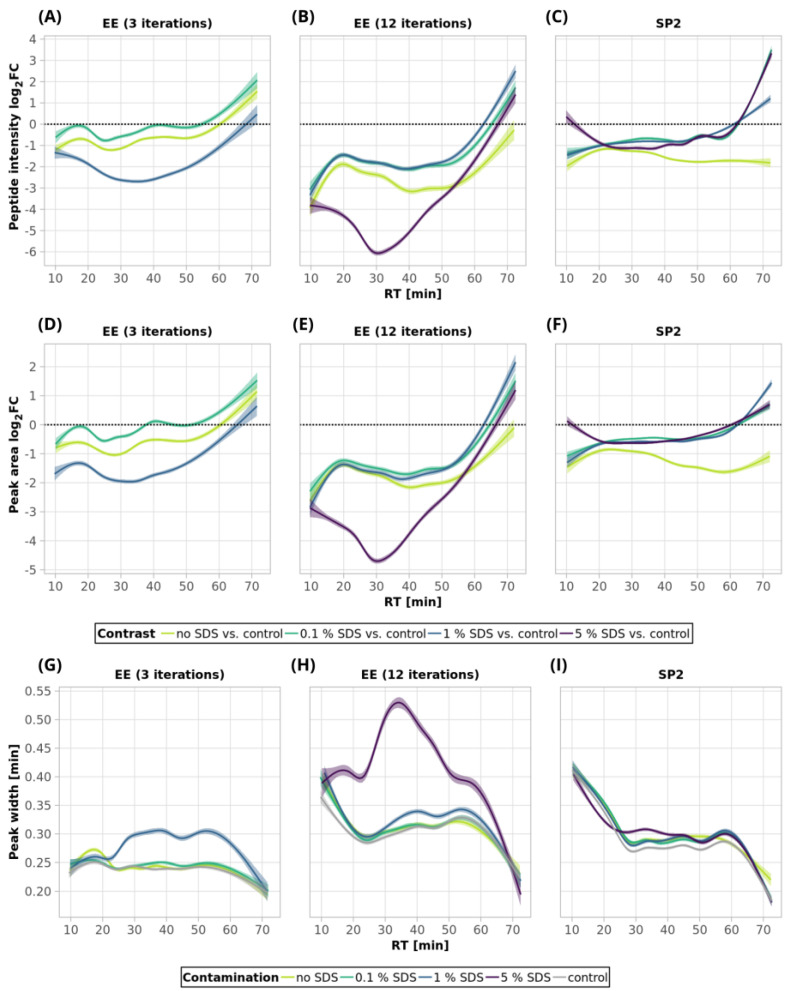
SDS clean-up of 250-ng peptide input by ethyl acetate extraction (3 and 12 iterations) and by SP2—smoothed trend lines (method GAM) of peptide intensity fold changes (log_2_ transformed, calculated from the comparison of the given condition vs. control, (**A**–**C**)), peptide area fold changes (log_2_ transformed, (**D**–**F**)), and peak widths (**G**–**I**). Only peptides quantified in at least 2 replicates out of 3 in either the control or a sample were used for the fold change calculation. Missing values were imputed by a local minimum. The confidence intervals (level 0.95) are represented by the shaded area alongside the lines. The black dotted lines denote no change (i.e., fold change = 1, log_2_FC = 0). See Materials and Methods for more details regarding the GAM curves. For scatterplots depicting individual datapoints of panels (**A**–**C**), see Figure S1. See Figure 1 for the experiments’ description.

**Figure 4:**
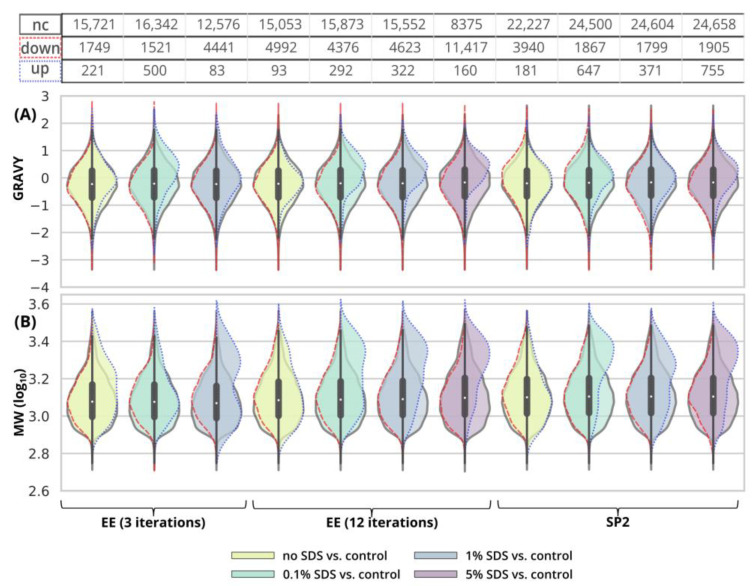
Distribution of GRAVY indexes (**A**) and MW (**B**) of peptides after EE and SP2 clean-up of 250-ng peptide input contaminated with SDS. Violin plots in the background represent GRAVY indexes or MW (log_10_) for peptides classified as not-changed after the clean-up (“nc”, black solid line), i.e., −3.333 ≤ log_2_FC ≥ 3.333 in the given comparison. The split violin plots on top of that represent the distribution of GRAVY or MW for peptides with decreased intensity (“down”, red dashed line, log_2_FC ≤ −3.333) and peptides with increased intensity (“up”, blue dotted line, log_2_FC ≥ 3.333). The width of all violin plots is the same, not based on the number of peptides. The numbers of observed peptides in each comparison are shown in the table above the violin plots. The colors of the violin plots’ filling represent the concentration of the contaminant. For scatterplots depicting individual datapoints, see Figures S2 and S3.

Using three EE iterations for the removal of 0.1% SDS contamination from a 250-ng peptide input sample was sufficient, and resulted in even more identified peptides (Figure 1A, Table S1) and lower quantitative losses compared to the processing of the uncontaminated sample (Figure 3A, green and light-green lines; detailed scatterplots for individual GAM curves can be seen in Figure S1). We lost only 6.6% of the identified peptides and observed comparable mean sum intensity compared to the control sample (Figure 1A and Figure 2A, Table S1), and the peak width profiles were not distorted (Figure 3G, green and light-green GAM curves). We observed that the peptides with 10× higher intensity compared to the control have a higher GRAVY index (Figure 4A and Figure S2), but the distribution of the molecular weight level practically did not change (Figure 4B and Figure S3). However, three EE iterations proved to be insufficient for the removal of 1% SDS contamination. We lost about half of the peptides (42.7%, Figure 1A, Table S1), and, furthermore, most of the identified peptides had decreased intensities compared to the control (Figure 3A, blue line, Figure S1), and the mean sum intensity decreased by 42% (Figure 2A). The imperfect removal of 1% SDS is also documented by widening the peak widths (Figure 3G; compare blue—1% SDS and green—0.1% SDS lines), indicating disturbed chromatographic separation.

In an attempt to remove higher SDS contaminations, we applied 12 EE iterations, which we considered as an acceptable processing maximum, to 1% and 5% SDS concentrations. The removal of 1% SDS contamination from the 250-ng peptide input sample was satisfactory with respect to the chromatographic behavior (Figure 3H, blue line), but we observed qualitative and quantitative losses comparable to those obtained for the processing of the uncontaminated input sample Figure 1A and Figure 2A, Table S1). In the case of the removal of 5% SDS contamination, even 12 iterations of EE were not sufficient, as evident from the shifted elution profile of iRT peptides (not shown), as well as the increased peak widths in general (Figure 3H, magenta line). As a result, the number of detected peptides decreased by 62.6% and 46.1% compared to the control and processed uncontaminated samples under the given conditions, respectively (Figure 1A, Table S1). We observed a decrease in the mean intensities sum by 65.5% (Figure 2A) and corresponding high quantitative losses of individual peptides (Figure 3B, magenta line, Figure S1) compared to the control sample.

As SDS widens the peak widths, in addition, we evaluated the quantitative changes based on the peak areas and observed similar trends to those seen for the intensities (Figure 3D–F).

Since even 12 EE iterations cannot remove the 5% SDS contamination, and the procedure was accompanied by substantial losses in the number of identified peptides (Figure 1A, Table S1), we continued in search for the optimal number of EE iterations only for the removal of 1% SDS contamination. The results obtained from the initial experiment showed that three EE iterations are not sufficient for the removal of 1% SDS from 250-ng peptide input samples, so we have tested a higher number of iterations (six and nine iterations) in order to select the optimal conditions for the removal of 1% SDS from 250 ng of peptides. The chromatographic peaks narrowed, as the result of the successful SDS removal, already after the application of six EE iterations, with comparable losses as those seen for three EE iterations (Figure S4B). Nine EE iterations further slightly improved the peak widths (Figure S4B) but led to higher losses (Figure S4A).

In conclusion, three EE iterations were sufficient to remove contamination up to 0.1% SDS from 250-ng *A. thaliana* peptide samples (Figure 1A, Figure 2A and Figure 3, Table S1), and six EE iterations are recommended for the removal of contaminations up to 1% SDS. We have not found sufficient EE conditions for the removal of 5% SDS contamination.

**Figure 5:**
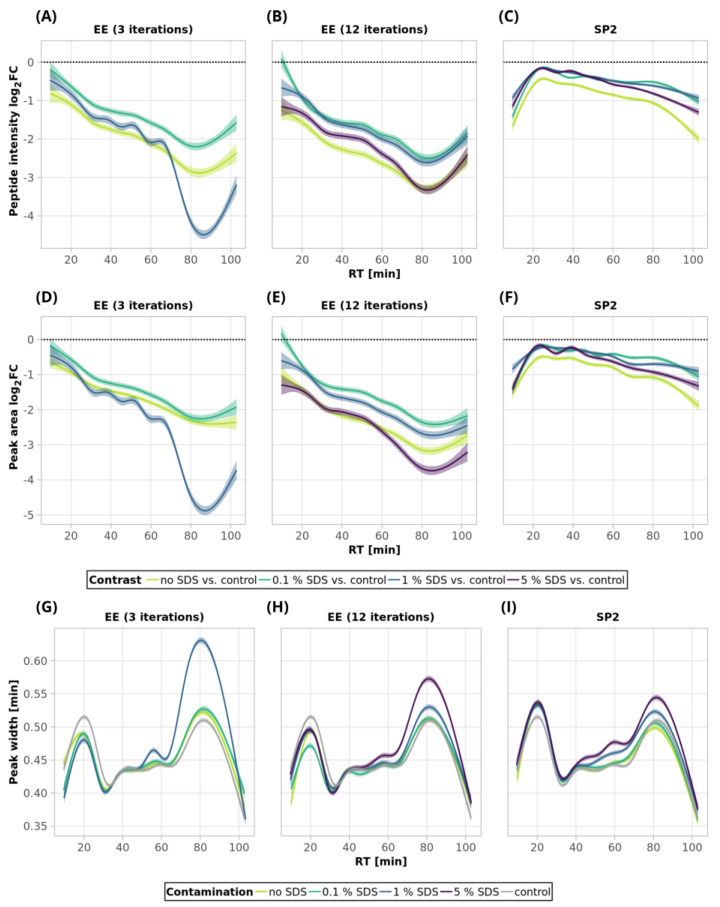
SDS clean-up of 10-µg peptide input by ethyl acetate extraction (3 and 12 iterations) and by SP2—smoothed trend lines (method GAM) of peptide intensity fold changes (log_2_ transformed, calculated from the comparison of the given condition vs. control, (**A**–**C**)), peptide area fold changes (log_2_ transformed, (**D**–**F**)), and peak widths (**G**–**I**). Only peptides quantified in at least 2 replicates out of 3, in either the control or a sample, were used for the fold change calculation. Missing values were imputed by a local minimum. The confidence intervals (level 0.95) are represented by the shaded area alongside the lines. The black dotted lines denote no change (i.e., fold change = 1, log_2_FC = 0). See Materials and Methods for more details regarding the GAM curves. For scatterplots depicting individual datapoints of the panels (**A**–**C**), see Figure S5. See Figure 1 for the experiments’ description.

In the case of 10 µg of uncontaminated peptide input, the number of identified peptides decreased by 16.8% after EE with 3 iterations and by 21.1% after 12 iterations (Figure 1B, Table S1). The sum intensity decreased by 29.5 and 40.3% after 3 and 12 EE iterations, respectively (Figure 2B, blue and green bars). Three EE iterations satisfactorily removed 0.1% SDS, the number of identified peptides decreased by 16.4%, and the mean sum of intensities was lower by 15% compared to the control. The peak widths are comparable to those of the control (Figure 5G, green line).

**Figure 6:**
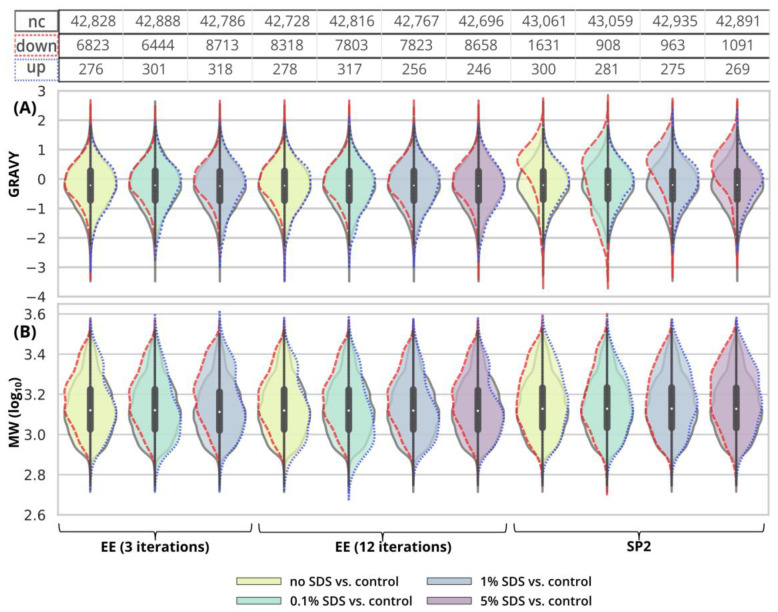
Distribution of GRAVY indexes (**A**) and MW (**B**) of peptides after EE and SP2 clean-up of 10-µg peptide input contaminated with SDS. Violin plots in the background represent GRAVY indexes or MW (log_10_) for peptides classified as not-changed after the clean-up (“nc”, black solid line), i.e., −3.333 ≤ log_2_FC ≥ 3.333 in the given comparison. The split violin plots on top of that represent the distribution of GRAVY or MW for peptides with decreased intensity (“down”, red dashed line, log_2_FC ≤ −3.333) and peptides with increased intensity (“up”, blue dotted line, log_2_FC ≥ 3.333). See Figure 4 for more information. For scatterplots depicting individual datapoints, see Figures S6 and S7.

The 1% SDS was the highest SDS concentration that we attempted to clean-up by three EE iterations in the case of the 10-µg input. The number of identified peptides decreased by 23.3% compared to the control, and the mean sum of peptide intensities decreased by 22.1% (Figure 1B and Figure 2B and Table S1). The peptides with the most decreased intensities were observed in the second half of the gradient, which correlates with increased peak widths (Figure 5A,G, blue line, and Figure S5). No distinct trends in hydrophobicity and molecular weight of peptides with reduced intensity were observed (Figure 6, Figures S6 and S7).

EE extraction with twelve iterations was tested for the clean-up of the 10-µg peptide input from the whole range of SDS contamination levels (0.1–5%). After the clean-up of 0.1%, 1%, and 5% SDS, we identified 19.8, 20.0, and 22.6% fewer peptides compared to the control, and the mean sum of intensities decreased by 23.1, 23.0, and 32.1%, respectively (Figure 1B and Figure 2B and Table S1). The same trends were observable not only in the intensities, but also in the peptides’ areas (Figure 5E). In the case of 5% SDS, the peak widths were slightly increased, but they are comparable to those of the control for the lower SDS concentrations (Figure 5H). We did not find any distinct trends in qualitative or quantitative behavior of the peptides concerning hydrophobicity or molecular weight (Figure 6, Figures S6 and S7).

Thus, EE extraction with 3 iterations can be applied for the clean-up of SDS contaminations up to 1% and with 12 iterations even up to 5%, with reasonable losses compared to those of the control for the 10-µg peptide input. We observed a positive effect of the presence of the SDS during EE on the suppression of losses caused by the clean-up step (compared to the uncontaminated sample), especially on the quantitative level (Figure 5A,B).

### 2.2. SDS Removal by SP2

After the initial optimization of the SP2 protocol in terms of solution volume and bead amount (see the final protocol in the Supplementary Material), we applied the SP2 procedure for the removal of SDS concentration up to 5% from both peptide inputs analogically, as for EE.

After the clean-up of the 250-ng peptide input without any contamination, we observed the loss of 21.3% of the identified peptides compared to the control peptide sample, and the average intensities decreased by 33.6% (Figure 1A and Figure 2A yellow bars, Table S1). The results from the analyses of the peptide solutions contaminated with 0.1% SDS showed similar trends to the EE clean-up process. SDS contamination, again, resulted in the reduction in quantitative losses (Figure 2A and Figure 3C and Figure S1) and more identified peptides compared to the processing of the uncontaminated sample (Figure 1A, Table S1). Specifically, we lost only 10.4% of the identified peptides, and the average intensity decreased only by 15.4% when the 250-ng peptide input sample was contaminated with 0.1% SDS. Moreover, hydrophobic peptides eluting at the end of the LC gradient displayed higher intensities compared to those in the analysis of the control sample (Figure 3C). Similar trends as those seen for 0.1% SDS contamination were observed also for 1% and 5% SDS. Comparable peaks’ widths for all contamination levels indicated complete removal of SDS from all of the contaminated 250-ng peptide input samples up to 5% concentration (Figure 3I).

Inspecting the data in terms of hydrophobicity (GRAVY index) and molecular weight revealed that, after SP2 treatment of the uncontaminated sample, we lost the peptides with a slightly decreased GRAVY index (Figure 4A and Figure S2), but the distribution of the molecular weight did not change (Figure 4B and Figure S3). As for the GRAVY indexes, we did not observe any distinct differences among the contamination levels; however, in the case of 5% SDS, there was a small population of peptides with increased intensities compared to the control group, with higher, more hydrophobic GRAVY values (Figure 4A and Figure S2). In the presence of SDS in the sample subjected to the SP2 protocol, the peptides with increased intensities compared to the control had a decreased molecular weight (Figure 4B and Figure S3).

The 10-µg input was processed in the same way via the SP2 protocol. We observed similar losses in terms of the number of identified peptides regardless of the presence or concentration levels of SDS (3.2% at most, Figure 1B, Table S1). However, the average peptide intensities sum decreased by 29.9% (Figure 2B) in the absence of the SDS in the sample. If the sample was contaminated with SDS, the intensity did not decline as dramatically (by 18.1% on average with the 5% SDS contamination at most, Figure 2B). The quantitative losses increased with the retention time (Figure 5C), and the same trend was also visible in the quantitative changes based on the area (Figure 5F). The minor chromatographic peaks widening compared to the control sample was observed in the samples contaminated with 5% and 1% SDS; however, the peak widths were narrower than the corresponding ones after the EE processing for either 3 or 12 iterations (Figure 5G,I; magenta lines).

After the SP2 treatment of the 10-µg input, we observed hydrophobic peptides with quantitative losses regardless of the presence or concentration of SDS in the sample (Figure 6A and Figure S6). We did not observe any major changes in molecular weight distribution (Figure 6B and Figure S7).

**Figure 7:**
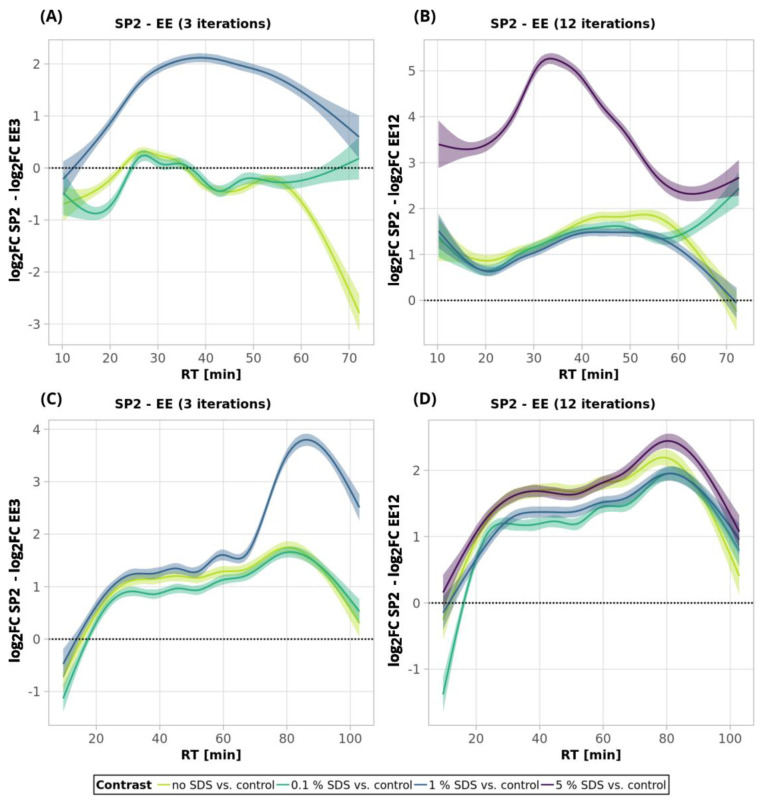
Comparison of ethyl acetate extraction (3 and 12 iterations) and SP2 for 250-ng (**A**,**B**) and 10-µg peptide inputs (**C**,**D**). Smoothed trend lines (method GAM) denote subtractions of peptide intensity log_2_FCs from SP2 and the log_2_FCs from the ethyl acetate extraction experiment (log_2_FC SP2–log_2_FC EE). The confidence intervals (level 0.95) are represented by the shaded area alongside the lines. The black dotted lines denote no change (i.e., fold change = 1, log_2_FC = 0). See Materials and Methods for more details regarding the GAM curves. See Figure 1 for the experiments’ description.

To summarize the comparison of the performance of ethyl acetate extraction and SP2 for SDS removal, we compared the changes in the intensities of individual peptides obtained using respective clean-up methods to the respective control (no contamination, no clean-up processing) experiment (Figure 7). The SP2 outperformed EE in all of the tested scenarios, except for the 250-ng peptide amount with no SDS contamination and three EE iterations. This might be explained by the generally higher peptide losses of the SP2 compared to the EE (three iterations).

### 2.3. PEG Removal by SP2

After SDS contamination, we applied the SP2 protocol for the removal of another common contamination—polyethylene glycol (PEG)—which cannot be removed by ethyl acetate extraction.

Three different concentrations (0.01, 0.1, and 1%) of a wide range of PEG polymer length mixtures (from PEG 200 to PEG 8000) were tested.

**Figure 8:**
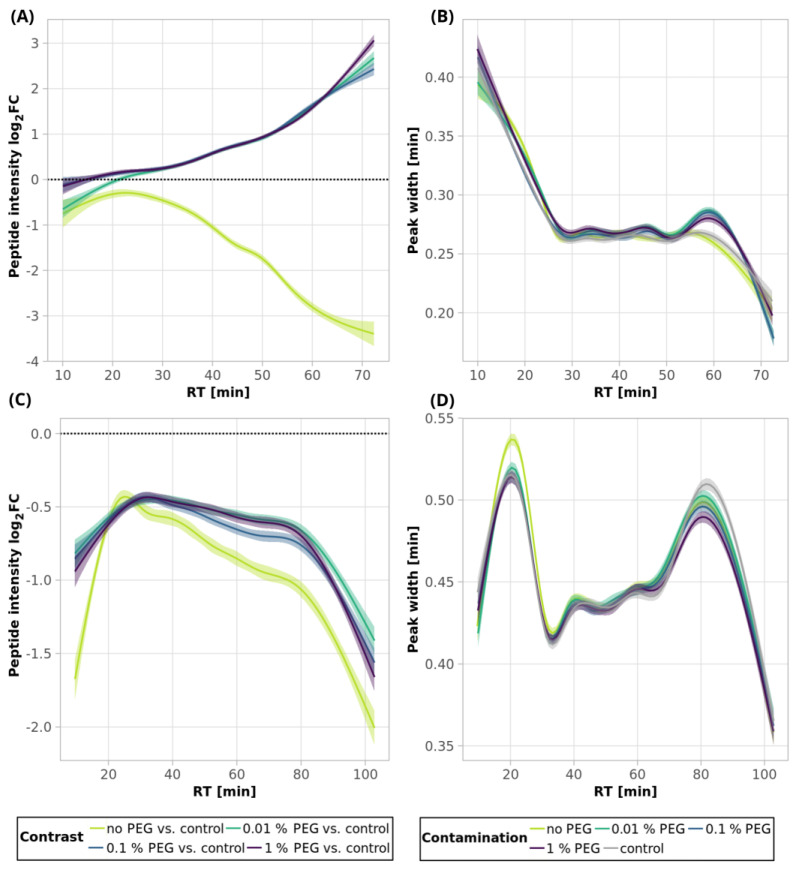
PEG clean-up of 250-ng (**A**,**B**) and 10-µg (**C**,**D**) peptide inputs by SP2—smoothed trend lines (method GAM) of peptide intensity fold changes (log_2_ transformed, calculated from the comparison of the given condition vs. control (**A**,**C**)); and peak widths (**B**,**D**). Only peptides quantified in at least 2 replicates out of 3, in either the control or a sample, were used for the fold change calculation. Missing values were imputed by a local minimum. The confidence intervals (level 0.95) are represented by the shaded area alongside the lines. The black dotted lines denote no change (i.e., fold change = 1, log_2_FC = 0). See Materials and Methods for more details regarding the GAM curves. For scatterplots depicting individual datapoints of panel A, see Figure S1; for panel C, see Figure S5. See Figure 1 for the experiments’ description.

**Figure 9:**
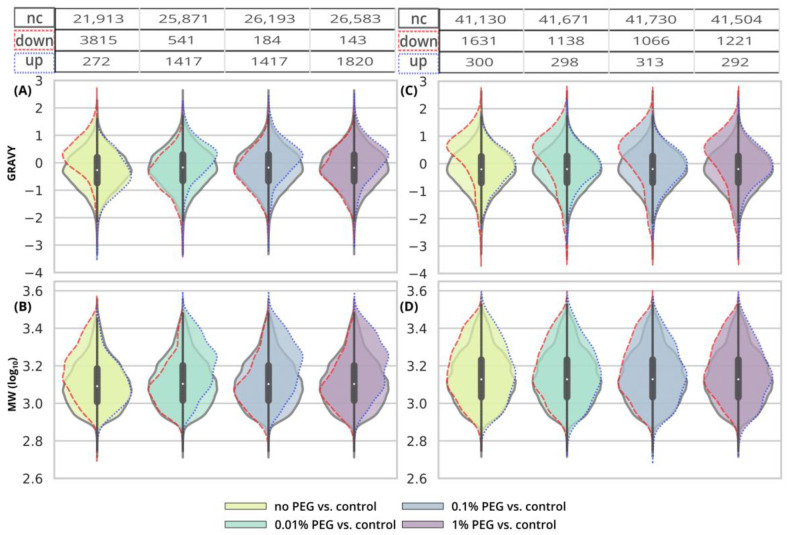
Distribution of GRAVY indexes (**A**,**C**) and MW (**B**,**D**) of peptides after SP2 clean-up of 250-ng (**A**,**B**) and 10-µg (**C**,**D**) peptide inputs contaminated with SDS. Violin plots in the background represent GRAVY indexes or MW (log_10_) for peptides classified as not-changed after the clean-up (“nc”, black solid line), i.e., −3.333 ≤ log_2_FC ≥ 3.333 in the given comparison. The split violin plots on top of that represent the distribution of GRAVY or MW for peptides with decreased intensity (“down”, red dashed line, log_2_FC ≤ −3.333) and peptides with increased intensity (“up”, blue dotted line, log_2_FC ≥ 3.333). See Figure 4 for more information. For scatterplots depicting individual datapoints, see Figures S9 and S12.

Similarly to the SDS contamination removal, we detected a decreased number of peptides (84.4%) after SP2 treatment of the uncontaminated sample with a lower mean sum intensity (87.1%) than that found for the control sample using 250-ng peptide input (Figure 1A and Figure 2A, Table S1). The quantitative losses increased throughout the retention time (Figure 8A, light-green line and Figure S8), which, together with the GRAVY value distribution (Figure 9A and Figure S9A), points to the existence of more hydrophobic peptides losses during the SP2 process.

However, we observed a higher number of detected peptides and mean quantities compared to the control sample in the presence of 0.01 and 0.1% PEG contamination in the sample followed by SP2 clean-up (Figure 1A and Figure 2A, orange bars, Table S1). The contamination of the sample by 1% PEG resulted in the reduction in detected peptides after the SP2 clean-up by 3.0%, compared to control (Figure 1A, Table S1). The beneficial effect of PEG on quantitative changes is reflected in Figure 8A and Figure S8, where it can be noticed that the peak intensities increase more for later eluting, thus showing more hydrophobic peptides. This is supported by the increased intensity dependence on GRAVY (Figure 9A and Figure S9A).

The beneficial effect of PEG contamination might be related to incomplete PEG removal, in agreement with our previous study on sample losses in LC-MS vials [19], despite the different PEG used in these studies. The previous study used single PEG 20,000, while here we have used a mixture of PEGs in order to better mimic various PEG contamination sources. We have indeed noticed the presence of some residual PEG after SP2 clean-up of the 250-ng peptide input samples with initial 0.1 and 1% PEG concentrations during the quality control analyses (Figure S10). Quality control analyses were run in a single-column setup (without the trap column), enabling better detectability of the PEG contamination in the on-line-connected mass spectrometer. Compared to the corresponding injection of PEG (0.01%), the residual PEG concentrations are estimated to be below 0.002%. As expected, based on our experience with PEG utilization to minimize sample losses [19], it is evident that the residual PEG in the sample did not influence the peak width (Figure 8B).

After the SP2 clean-up of the 10-µg peptides contaminated with all three concentrations of PEG, the number of identified peptides slightly decreased (less than 4%) compared to the unprocessed control sample (Figure 1B, orange bars, Table S1), as well as the mean sum of intensities (Figure 2B, orange bars). The quantitative losses were more evident with an increasing retention time (Figure 8C and Figure S11). However, the numbers of identified peptides are comparable to those obtained by SP2 processing of the uncontaminated sample, and the mean sums of intensities are even higher for all of the tested PEG concentrations (Figure 1B and Figure 2B, Table S1). The peak widths are comparable for all of the samples, regardless of clean-up processing (Figure 8D). We observed a small population of hydrophobic peptides with decreased intensities but without any distinct change in the distribution of molecular weight (Figure 9C,D and Figure S12).

### 2.4. SP2 Clean-Up of SDS and PEG Combined Contamination

Next, we tested the SP2 protocol for removing the combination of 5% SDS and 1% PEG (maximum applied concentrations).

After cleaning the contaminated sample containing 250 ng of peptides, we lost only 2.9% of the identified peptides, and the mean sum of intensities increased by 7.6% in comparison with the control (Table S1, Figure S13A). We observed both scenarios, including peptides with decreased (approximately 1st half of the LC gradient) as well as increased (approximately 2nd half of the LC gradient) intensities after the SP2 process (Figure S14A). The GRAVY indexes and molecular weight distributions are shown in Figure S15A,B. The peak widths are comparable to the control and the uncontaminated sample after SP2 (Figure S14B), but a bit wider, demonstrating close-to-complete SDS contamination removal.

In the case of the 10-µg peptide input, we identified 4.0% fewer peptides compared to the control, and the mean sum of intensities decreased by 16% (Table S1 and Figure S13B). We observed mainly peptides with decreased intensities, with the negative effect being more profound with an increasing retention time of the peptide (Figure S14C). The peak widths were mostly slightly increased compared to the control and uncontaminated sample after SP2 (Figure S14D), suggesting higher residual SDS contamination compared to the 250-ng peptide variant. The distribution of intensity log_2_FC values based on the GRAVY indexes showed decreased intensities mainly for the more hydrophobic peptides, with no apparent trend in MW (Figure S15C,D), a slightly different situation compared to the 10-µg input, where the same peptide categories showed increased intensities (Figure S15A,B).

### 2.5. SP2 Clean-Up of 10-ng Peptide Input Sample

In order to evaluate the applicability of our SP2 protocol to lower the peptide input amounts used routinely on highly sensitive instrumentation, we tested this method on a 10-ng peptide input. We tried to clean up the sample individually from SDS (0.1 and 1%) and PEG (0.01 and 0.1%) contamination.

**Figure 10:**
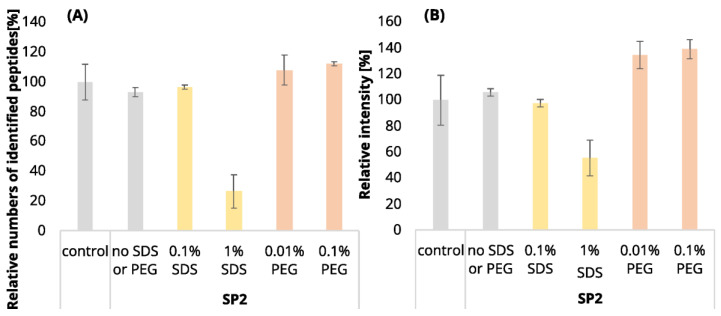
SP2 clean-up of 10-ng peptide input contaminated with SDS or PEG. Mean numbers of identified peptides (**A**) and mean intensity sums of quantified peptides (**B**) compared to the control (100%). The standard deviations are displayed as error bars. The gray color represents uncontaminated samples (control and SP2 treated), the yellow color represents samples contaminated by SDS after SP2 clean-up, the orange color represents samples contaminated by PEG after SP2 clean-up. See Table S2 for more details regarding the numbers of identified peptides.

**Figure 11:**
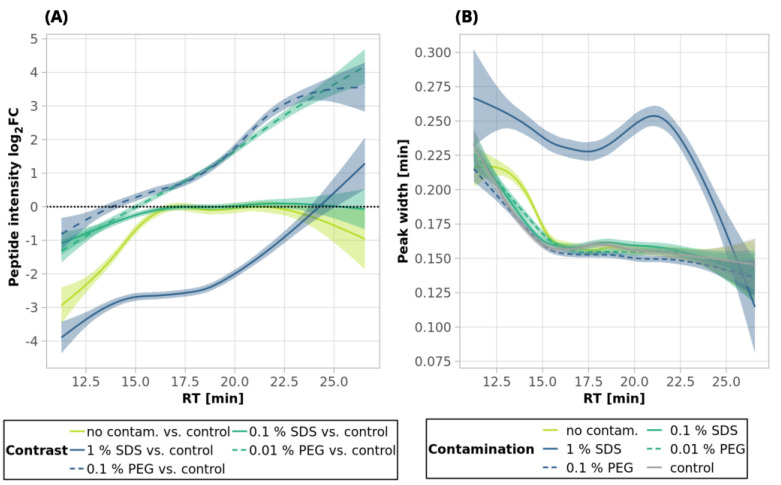
SDS and PEG clean-up of 10-ng peptide input by SP2—smoothed trend lines (method GAM) of peptide intensity fold changes (log_2_ transformed, calculated from the comparison of the given condition vs. the control (**A**)), and peak widths (**B**). Only peptides quantified in either at least 1 replicate of the control (only 2 replicates available) or 2 out of 3 replicates of a sample were used for the fold change calculation. Missing values were imputed by a local minimum. The confidence intervals (level 0.95) are represented by the shaded area alongside the lines. The black dotted lines denote no change (i.e., fold change = 1, log_2_FC = 0). See Materials and Methods for more details regarding the GAM curves.

After the SP2 treatment of the uncontaminated sample, we lost 6.9% of the identified peptides (Figure 10A, Table S2), and the mean sum of intensity increased by 5.6% (Figure 10B). The SP2 clean-up of the 10-ng peptide sample contaminated with 0.1% SDS resulted in the loss of 3.5% of the identified peptides, and the mean sum of intensities decreased by only 2.4% (Figure 10A,B, Table S2). The peak widths are comparable to the control and the uncontaminated samples after the clean-up (Figure 11B, green and light-green continuous lines). However, in the case of 1% SDS, we encountered the limits of the protocol. We observed a loss of 73.6% of the identified peptides, and the mean sum of intensities decreased by 44.7% (Figure 10A,B, Table S2). There were quantitative losses throughout the gradient (Figure 11A, blue continuous line), and the peak widths increased, which indicates presence of the SDS in the sample even after the SP2 clean-up (Figure 11B, continuous blue line).

After the SP2 clean-up of the 10-ng peptide input contaminated with 0.01 and 0.1% PEG, we identified 7.9 and 12.4% more peptides compared to the control, respectively (Figure 10A, Table S2). The intensities increased by 34.8 and 39.1%, respectively (Figure 10B). The increase in intensities is observable for the majority of peptides throughout the gradient, but increases with increasing retention time (Figure 11A, dashed lines). The peak widths are comparable to the control and the cleaned-up uncontaminated samples, as expected (Figure 11B, dashed lines).

## 3. Discussion

Sample preparation for bottom-up proteomics is a multistep procedure and only the optimal performance of each step ensures obtaining reliable maximum information about the analyzed sample. 

In this study, we focused on the clean-up of plant peptide samples. Plant samples require relatively strong conditions for complete lysis, including the application of a high concentration of detergents. Thus, some residual contamination might easily appear in the final peptide solution to be analyzed on the LC-MS platform, interfering with protein identification and quantification. We selected *A. thaliana* leaves as the model plant sample and SDS and a mixture of different PEGs as the common contaminants. For SDS removal in the concentration range of 0.1–5%, we tested two clean-up methods—the performance of ethyl acetate extraction (EE) was compared to the SP2 protocol on carboxylate-modified paramagnetic beads. Next, only the SP2 protocol was evaluated for the removal of PEGs in the concentration range of 0.01–1%, because ethyl acetate extraction is not by principle suitable for the clean-up of this type of contaminant.

In the case of EE, we followed the protocol reported by Yeung and Stanley [17]. The main optimized parameter was the number of EE iterations. The SP2 protocol [12] was optimized and applied for all of the experiments under universal conditions, which are described in detail in the Supplementary Material, with no need to adjust any of the parameters based on the input material, type, or the concentration of the contaminations. The contamination experiments were performed over a wide range of peptide input amounts—from 10 ng to 10 µg.

In summary, we found the conditions for the sufficient removal of selected contaminants that enabled undisturbed LC-MS/MS analysis, except for EE clean-up of the 250-ng peptide input sample contaminated with 5% SDS, which represented quite an extreme level of contamination. Next, the SP2 clean-up procedure showed superior performance to the EE under the tested conditions. In the case of SDS removal, the SP2 outperformed the EE on both qualitative and quantitative levels of peptide characterization. In both of the tested peptide inputs (250 ng and 10 µg), the SP2 provided lower qualitative and quantitative losses than the ethyl acetate extraction, especially with an increased SDS concentration in a sample. After the SP2, we observed more peptides compared to when EE was applied. Chromatographic separation performed better after SP2 clean-up, too, as we observed narrower peptide peaks even in the presence of the highest SDS concentration, as opposed to the EE protocol. However, we noticed decreased intensities of peptides with higher GRAVY indexes after the SP2 of the 10-µg peptide samples, suggesting the loss of hydrophobic peptides during the clean-up. The proposed SP2 protocol offers the advantage of unified conditions for both of the tested contaminants—SDS up to 5% and PEGs up to 1% concentrations, and even for the simultaneous removal of both (tested for 250-ng and 10-µg peptide inputs). The other general advantages of SP2 are its rapidness and possibility for automation [12]. On the other hand, the initial peptide sample volume is limited to only 15 µL in the SP2 protocol; therefore, sample volume reduction, accompanied by the danger of the concentration of the contaminants, can be required. SP2 also requires additional accessories such as a magnetic rack and beads. In the case of EE, the number of EE iterations should be optimized for different SDS concentrations and peptide amounts in order to prevent extra losses, which might be difficult, as the level of contamination is usually unknown. The potential of both of these methods to remove various concentrations of SDS or PEG is summarized in Table 1. The capability of the SP2 to remove both of the tested contaminants and the usage of the unified protocol make this method the preferred method for clean-up prior to LC-MS/MS. 

Finally, the proposed SP2 protocol was applied for the clean-up of a 10-ng sample. The protocol was efficient for the removal 0.1% SDS contamination only. The PEG contamination up to 0.1% was not completely removed either, but PEG residuals in the sample did not negatively influence the LC-MS/MS analysis.

The SP2 clean-up procedure causes relatively high losses when applied to the uncontaminated sample. However, the qualitative and quantitative losses are substantially reduced, or even better results are obtained, in the presence of contamination (compared to the analysis of the control sample), especially in the presence of low levels of PEGs. This correlates with the results of our previous study dealing with peptide losses during their storage in autosampler vials [19], where we showed that the addition of PEG 20,000 in a final concentration of 0.001% to samples prepared for LC-MS/MS analysis originated from *A. thaliana* suppressed the losses up to 48 h using the LC setup with precolumn.

## 4. Materials and Methods

### 4.1. Plant Material and Growth Conditions 

*Arabidopsis thaliana*, ecotype Columbia 0, plants were grown in soil. Rosette leaves were harvested 4 weeks after planting and were ground in liquid nitrogen in a Freezer/Mill 6870 (SPEX SamplePrep; Metuchen, NJ, USA) in 3 cycles (2 min grinding and 2 min cooling per cycle) to obtain a powder.

### 4.2. Protein Extraction 

SDT buffer (4% SDS, 100 mM DTT; 100 mM Tris/HCl, pH 7.6) was used for lysis. An aliquot of 1 g of frozen AT plant tissue powder was solubilized in a Thermo Mixer C (Eppendorf; Hamburg, Germany) in 5 mL of hot SDT buffer for 30 min at 95 °C and 1000 rpm. To remove the insoluble material from the sample, the extract was centrifuged at room temperature for 10 min at 20,000× *g*. 

### 4.3. Tryptophan Fluorescence Assay

For the total protein concentration measurements, an assay based on the fluorescence spectrometry of tryptophan in a buffer containing 8 M urea in 20 mM Tris/HCl, pH 7.6 [20], was performed on a Cary Eclipse fluorescence spectrophotometer (Agilent Technologies; Santa Clara, CA, USA) in triplicate. The linear calibration curve was measured in the range of tryptophan concentrations from 0.0625 to 0.5 ng/µL (R^2^ = 0.9999). The fluorescence was excited at 295 nm and measured at 350 nm for emission in measurement window 310–450 nm, and the slits were set to 5 nm. 

### 4.4. Filter-Aided Peptide Sample Preparation (FASP)

The FASP procedure was performed according to Wiśniewski et al. [1], with some modifications. The extracted proteins in the SDT buffer were 5× diluted with urea buffer (8 M urea in 0.1 M Tris/HCl, pH 8.5) and transferred to centrifugal filter units Microcon 30 kDa (MRCF0R030, Merck; Darmstadt, Germany), washed 5 times in total (instead of 3 standard washing steps) with urea buffer to improve the residual SDS removal, and alkylated with 0.5 M iodoacetamide in urea buffer. The urea buffer was then replaced with 50 mM ammonium bicarbonate (ABC). The proteins were digested with trypsin (an enzyme-to-protein ratio 1:50) and incubated for 18 h at 37 °C. The resulting peptides were recovered by centrifugation and two filter washes using 50 mM ABC buffer. All centrifugation steps were carried out at 14,000× *g*, except for the protein loading steps (7000× *g*). The peptides were not subjected to any cleaning step to avoid interference with the following SP2 and EE procedures (e.g., losing some set of peptides). The detailed FASP protocol can be found in the Supplementary Materials.

### 4.5. Contamination of Peptides

The tryptic peptides from the FASP step were subsequently concentrated using the SpeedVac system (Thermo Fisher Scientific; Waltham, MA, USA) to volume, allowing us to collect 15-μL aliquots containing the requested quantity of peptides, and contaminated with various concentrations of SDS (up to 5%) or PEG (up to 1%). A total of 2% PEG stock solution for the contamination was prepared by mixing the same volumes of PEG 200, 400, and 600 (TCI; Tokyo, Japan); and PEG 3000 (Merck), 4000 (Carl Roth; Karlsruhe, Germany), and 8000 (Merck) stock solutions in water to achieve the same *v*/*v* concentration of each PEG component in the final solution. One contaminated solution with the specified contamination combination was prepared and aliquoted for further parallel and repeated (*n* = 3) processing using the selected clean-up procedures.

### 4.6. SP2 Peptide Clean-Up Protocol

The SP2 protocol used in this study is based on recommendations published in Waas et al. and Wojtkiewicz et al., [12,13]. All experiments were carried out in triplicate using the identical peptide solution with or without specified contamination addition. The peptide clean-up was performed in polypropylene 2-mL microtubes with a V-shaped bottom. A detailed and optimized step-by-step SP2 protocol is described in the Supplementary Material. In contrast to the previously published protocols [12,13], there is no need to adjust the amount of beads and working volumes to the amount of peptides (tested range of 10 ng to 10 µg) in our protocol. 

Carboxylate-modified paramagnetic beads stock was prepared by mixing 1:1 (*v*/*v*) of two types of carboxylate-modified Sera-Mag SpeedBeads (Cytiva; Marlborough, MA, USA): hydrophilic solids (cat. no. 45152105050250) and hydrophobic solids (cat. no. 65152105050250). The mixture of magnetic beads was washed and resuspended in MilliQ water (stock beads concentration 50 μg/μL). A Pure Proteome magnetic stand (Merck, cat.no. LSKMAGS08) was used for sample processing. 

Binding: The carboxylate-modified paramagnetic beads stock was mixed thoroughly by shaking to ensure that the beads were entirely suspended in the solution. The exact volume of 6 μL of beads suspension was added to 15 μL of peptide sample and subsequently mixed by repeated pipetting. Acetonitrile 100% (399 μL) was added to each sample to obtain 95% acetonitrile concentration. The samples were mixed by repeated pipetting and allowed to settle for 5 min on the bench before being placed on the magnetic stand for an additional 2 min. The supernatant was removed with a pipette. 

Washing: The tubes were removed from the magnetic stand and 500 μL of 100% acetonitrile was added to each tube. The samples were mixed by repeated pipetting and allowed to settle for 2 min on the bench before being placed on the magnetic stand for an additional 2 min. The supernatant was removed with a pipette. The acetonitrile wash step was repeated once more.

Elution: The tube was removed from the magnetic stand, and the beads were reconstituted in 54 μL of 2% acetonitrile in water. The samples were vortexed for 30 s and allowed to settle for 2 min on the bench before brief centrifugation. Then, the tubes were placed on the magnetic stand for an additional 2 min. The supernatant containing the cleaned peptides was transferred with a pipette into clean 0.5-mL microtubes. The elution step was repeated once more, and the second eluate was added to the first one. Combined volumes of the cleaned peptides were centrifuged at 20,000× *g* for 10 min, and the supernatant was transferred with a pipette into a new 0.5-mL microtube. 

### 4.7. Ethyl Acetate Extraction

All experiments were carried out based on the protocol published by Yeung and Stanley [17] and carried out in triplicate. A detailed step-by-step ethyl acetate extraction protocol is described in the Supplementary Material. The peptide samples were diluted with 50 mM ammonium bicarbonate to the final volume of 150 μL. Water-saturated ethyl acetate (1 mL) was added to each sample, which was then shaken vigorously in a Thermomixer at 2000 rpm at room temperature for 2 min. Phases were separated by centrifugation at 19,000× *g* at room temperature for 1 min. The upper ethyl acetate layer was aspirated and discarded. The extraction was performed in several iterations (see the main text for more details). The resulting bottom aqueous phase containing cleaned peptides was evaporated on a SpeedVac vacuum concentrator (Thermo Fisher Scientific).

### 4.8. Acidic Extraction

Dried peptides were extracted prior to analysis in the following two steps: at first, with 25 µL of 5% formic acid (FA) and 25 µL of 100% acetonitrile (ACN), and after that with 100 µL of 100% ACN. Both solutions were pooled into an LC-MS vial with already added polyethylene glycol (20,000; PEG) [19]. The samples were concentrated in a SpeedVac to remove ACN (volume after concentrating about 10 µL), and the sample volume was adjusted to 15 µL using MilliQ water (final PEG concentration 0.001%). 

### 4.9. Peptide Quality Control Analyses after FASP

The peptide concentration estimation and quality control (QC) before the final LC-MS/MS analyses were carried out with an Ultimate 3000 RSLCnano system (Thermo Fisher Scientific) on-line-connected to an Impact II Ultra-High Resolution Qq-Time-Of-Flight mass spectrometer (Bruker; Bremen, Germany). The peptides were directly loaded and separated on the PepMap C18 analytical column (2 µm particles, 300 µm × 15 cm, 40 °C; Thermo Fisher Scientific) using 2% of mobile phase B (0–5 min, 10 μL/min; mobile phase A: 0.1% FA in water, mobile phase B: 0.1% FA in 80% acetonitrile), for the effective gradient mobile phase B increased from 2 to 60% (5–25 min, 6 μL/min). After that, mobile phase B increased to 95% in 1 minute and remained at this state for the next 4 min (26–30 min, 10 μL/min). Finally, the column was equilibrated for 5 min with 2% mobile phase B. The analytical column outlet was connected to the ESI Apollo sprayer ion source (Bruker). MS and MS/MS spectra were measured in the data-dependent mode with a 2.5 s long cycle. The *m/z* mass range was set to 110–2000 *m/z*, with precursor selection from 300 *m/z* to 2000 *m/z*. The measurement frequencies of the MS and MS/MS scans were 2 Hz and 4–16 Hz (depending on the precursor intensity), respectively. The system was externally calibrated using Pierce HeLa digest (PN 88329), with the calibration curve ranging from 5 to 200 ng per injection. The area under the total ion chromatogram trace (excluding the washing part of the analysis, 7–28th min) was used for the calibration curve preparation and for the QC analyses results evaluation. 

### 4.10. LC-MS/MS Analysis of Peptides

The LC-MS/MS analyses of all peptide mixtures were performed using an Ultimate 3000 RSLCnano system connected to an Orbitrap Exploris 480 mass spectrometer (Thermo Fisher Scientific). We injected 6.5/15 of the peptide solution in the case of the 10- and 250-ng inputs and a volume corresponding to 1 µg in the case of the 10-µg input (based on the QC analyses, see above). Prior to LC separation, tryptic digests were on-line-concentrated and desalted using a trapping column (300 μm × 5 mm, μPrecolumn, 5μm particles, Acclaim PepMap100 C18, Thermo Fisher Scientific; temperature of 40 °C). After the washing of the trapping column with 0.1% FA, the peptides were eluted (flow rate: 300 nL/min) from the trapping column to an analytical column (EASY spray column, Acclaim Pepmap100 C18, 2 µm particles, 75 μm × 250 mm; Thermo Fisher Scientific) by a 15, 60, or 90 min linear gradient program (5–37% of mobile phase B; mobile phase A: 0.1% FA in water; mobile phase B: 0.1% FA in 80% ACN) for the 10-ng, 250-ng, and 10-µg peptide samples, respectively, followed by a 10-min wash at 80% of mobile phase B. Equilibration of the trapping column and the analytical column was carried out prior to sample injection to sample loop. The analytical column was installed in the EASY-Spray ion source (Thermo Fisher Scientific) according to the manufacturer’s instructions, with a column temperature of 40 °C. The spray voltage and sweep gas were set to 1.9 kV and 1 a.u., respectively.

Data were acquired in a data-independent acquisition mode (DIA). The survey scan covered an *m/z* range of 350–1400 at the resolution of 60,000 (at *m/z* 200) and a maximum injection time of 55 ms. HCD MS/MS (27% relative fragmentation energy) were acquired in the range of *m/z* 200–2000 at 30,000 resolution (maximum injection time 55 ms). Overlapping window schemes in the *m/z* range from 400 to 1000 were used as isolation window placements (see transitions_list.xlsx file for more details).

### 4.11. Data Analysis

The acquired raw DIA data were demultiplexed and converted to mzML files with the MSconvert tool (version: 3.0.21193-ccb3e0136, developer build). The mzML files were processed in DIA-NN version 1.8.0 [21] using a project-specific DIA data-based library.

Libraries based on the FASTA protein databases were generated at first using a modified cRAP database (based on http://www.thegpm.org/crap/; accessed on 22 November 2018; 111 sequences in total) and UniProtKB protein database for *Arabidopsis thaliana* (https://ftp.uniprot.org/pub/databases/uniprot/current_release/knowledgebase/reference_proteomes/Eukaryota/UP000006548/UP000006548_3702.fasta.gz, accessed on 10 December 2021; version 2021/11, number of protein sequences: 27,469). Protein inference was set to Isoform IDs. No optional, carbamidomethylation as fixed modification and trypsin/P enzyme with 1 allowed missed cleavage, peptide length 7–30, charge 1–4, *m/z* range according to the data acquisition parameters (precursor and fragments *m/z* ranges: 350–1050 and 200–2000, respectively) were set during the FASTA-files-based library preparation step. 

A project-specific library was created using a DIA-NN search utilizing all DIA analyses of the 10-µg sample variants using the FASTA-files-based library. The false discovery rate (FDR) control was set to 1% FDR. The MS1 and MS2 accuracies, as well as scan window parameters, were set based on the initial test searches (median value from all samples ascertained the parameter values). 

The final search was performed using the project-specific library built in the previous step. The algorithm settings were the same as those used for the project-specific library preparation step. The MBR option was checked, but the results from the first pass were used for further processing. See the provided DIA-NN log files for more details.

The DIA-NN main output files without MBR were further processed using the software container environment (https://github.com/OmicsWorkflows/KNIME_docker_vnc, version 4.1.3a, accessed on 10 December 2021). Briefly, it covered the following: (1) filtering out of precursors with Global.Q.Value ≥ 0.01 and Quantity.Quality ≤ 0.5; (2) calculating peak widths as the subtraction of the precursors peaks’ START and STOP retention times; (3) precursor level long table pivoting to the wide table format and grouping to the stripped peptide sequence level (taking the maximum precursor intensities, peak areas, retention times, and peak widths); (4) removal of the sequences associated with the contaminant protein groups (e.g., cRAP proteins); (5) normalization of the intensities and peak areas according to the injection volume (only in the case of the 10-µg peptide input); (6) peptide intensities and peak areas log_2_ transformation; (7) median intensities and peak areas calculation using the log_2_-transformed technical replicate results; (8) de-log_2_ transformation of the median intensities and areas; (9) calculating the de-logged median intensities and peak area fold changes—only for the peptides quantified in at least 2 replicates out of 3 in either the control or a sample (or in at least 1 replicate if only 2 replicates were available)—imputing pairwise missing values by a local minimum; (10) log_2_ transformation of fold changes; and (11) visualization of selected data. See the provided workflow for more details.

## 5. Conclusions

We performed a comparison of two clean-up techniques for the removal of SDS contamination from *A. thaliana* tryptic peptide samples. While ethyl acetate extraction was able to efficiently remove SDS contamination of up to 1% under the applied conditions (Table 1), our optimized SP2 protocol efficiently removed even 5% SDS contamination from 250-ng and 10-µg peptide inputs. Our results indicate that the SP2 technique can perform effectively across a wide range of beads and peptide ratios, and, thus, the protocol can be used without any adjustments for a wide span of peptide inputs, similarly to our previously reported SP3 protocol [22]. For the tested peptide inputs, the SP2 protocol proved to be satisfactory for the removal of PEG contamination up to 1%, and even for simultaneous SDS (5%) and PEG (1%) contamination. In contrast, the addition of a low concentration of PEG might eliminate the losses during the clean-up. With a decreased peptide input to 10 ng, the ability of our SP2 protocol to remove SDS decreased to 0.1% concentration. The PEG contamination levels (up to 0.1%) even caused qualitative and quantitative gains for the 10-ng peptide input, implying the possibility of the intentional addition of PEG prior to the SP2 clean-up step.

## Figures and Tables

**Table 1:** Summary of the recommended clean-up methods for 250-ng and 10-µg peptide inputs contaminated with SDS and PEG.

		Peptide Input	
Contamination		250 ng	10 µg
**SDS**	0.1%	EE-3/SP2	EE-3/SP2
	1%	EE-6/SP2	EE-3/SP2
	5%	SP2	SP2
**PEG**	0.01–1%	SP2	SP2

## Data Availability

The mass spectrometry proteomics data have been deposited to the ProteomeXchange Consortium via the PRIDE [23] partner repository with the dataset identifier PXD045031.

## Supplementary Materials

The following supporting information can be downloaded at: https://www.mdpi.com/article/10.3390/ijms242417347/s1.
