# Supplementary material for: Benchmarking of Two Peptide Clean-Up Protocols: SP2 and Ethyl Acetate Extraction for Sodium Dodecyl Sulfate or Polyethylene Glycol Removal from Plant Samples before LC-MS/MS
Source: Int J Mol Sci. 2023 Dec 11;24(24):17347. doi: 10.3390/ijms242417347 (PMC10743447; doi:10.3390/ijms242417347)
Supplement: Supplementary file 1 [file ijms-24-17347-s001.zip › ijms-2737788-supplementary.pdf]

SP2 Clean-up of SDS or PEG from Plant Samples before LC-MS/MS

Petra Martinková[1,2], Hana Konečná[1,2], Petr Gintar[1,2], Karolína Kryštofová[1,2], David Potěšil[1], Martin Trtílek[3], Zbyněk Zdráhal[1,2*]

[1]Mendel Centre for Plant Genomics and Proteomics, Central European Institute of Technology, Masaryk University, Kamenice 5, 625 00 Brno, Czech Republic

[2]National Centre for Biomolecular Research, Faculty of Science, Masaryk University, Kamenice 5, 625 00 Brno, Czech Republic

[3]Photon Systems Instruments, Spol. s r.o., Průmyslová 470, 664 24 Drásov, Czech Republic
[*]Correspondence: zdrahal@sci.muni.cz

LIST OF SUPPLEMENTARY MATERIAL

Materials and Methods

1) Filter-Aided Sample Preparation (FASP)
2) Contaminating Stocks
3) Ethyl Acetate Extraction
4) SP2 Peptide Universal Clean-Up Protocol

Tables and Figures

Table S1
Figure S1
Figure S2
Figure S3
Figure S4
Figure S5
Figure S6
Figure S7
Figure S8
Figure S9
Figure S10
Figure S11
Figure S12
Figure S13
Figure S14
Figure S15
Table S2

FILTER-AIDED SAMPLE PREPARATION (FASP)

Materials
Centrifugal filter units Microcon 30 kDa (MRCF0R030, Merck)
ThermoMixer C (Eppendorf)
Savant SpeedVac SPD130DLX Vacuum Concentrator (ThermoFisher Scientific)
Incubator – set to 37°C (Memmert IF30; Schwabach, Germany)

Solutions
Urea buffer: 8 M urea in 0.1 M Tris/HCl, pH 8.5
0.5 M iodoacetamide (IAA) in urea buffer
50 mM ammonium bicarbonate (ABC) in water
SOLu-Trypsin (EMS0004, Merck)

Procedure

- Mix 60 µL of a protein extract in SDT buffer with 400 µL of urea buffer and load onto the centrifugal filter units and centrifuge at 7000× *g* for ca. 15 min
- Add 200 µL of urea buffer, centrifuge at 14,000× *g* for ca. 15 min
- Add 100 µL of urea buffer with IAA, mix in dark at 900 rpm in a ThermoMixer for 1.5 min at RT and then 20 min at 350 rpm (RT), centrifuge at 14,000× *g* for ca. 10 min
- Wash by adding 100 µL of urea buffer, centrifuge at 14,000× *g* for ca. 10 min, repeat this step 4 times (5 times total)
- Wash 3 times by adding 100 µL 50 mM ABC, centrifuge at 14,000× *g* for ca. 10 min
- Add trypsin in 50 mM ABC (total volume 50 µL) to have enzyme to protein ratio 1:100
- Incubate the filter units covered with parafilm (to avoid evaporation of the solution from the unit) in a wet chamber in incubator at 37 °C for 18 hours
- Remove the parafilm and transfer the filter units to new collection tubes
- Centrifuge the filter units at 14,000× *g* for ca. 10 min
- Add 50 µL of 50 mM ABC and centrifuge the filter units at 14,000× *g* for ca. 10 min, repeat this step once (a total of 2 times), the final volume of FASP eluate is ca. 150 µL

CONTAMINATING STOCKS

SDS stock 10% in water (w/v)
PEG stock 2% in water: mix the same volumes of 2% PEG 200, 400, and 600 (TCI); and PEG 3000 (Merck), 4000 (Roth), and 8000 (Merck)

ETHYL ACETATE EXTRACTION PROTOCOL

Materials
Ethyl acetate (HPLC grade, 99.7%, Merck)
Milli-Q water
50 mM ammonium bicarbonate
ThermoMixer C (Eppendorf)
Savant SpeedVac SPD130DLX Vacuum Concentrator (ThermoFisher Scientific)

Preparation of water-saturated ethyl acetate
For cleaning of contaminated tryptic peptides, water-saturated ethyl acetate was prepared. Approximately 80 mL of ethyl acetate was mixed with approximately 10 mL of Milli-Q water in a glass volumetric flask. The flask was closed with a glass stopper, and shaken vigorously for 1 minute, then the mixture rested for 5 minutes. The shaking was repeated twice (three times in total). After the third shaking cycle, the mixture was transferred into a glass reagent bottle with a screw cap and let rest for at least 30 minutes at room temperature in the dark until the clear ethyl acetate upper phase was formed.

Extraction procedure
Peptide samples were diluted by 50 mM ABC to the final volume of 150 µL. 1 mL of water-saturated ethyl acetate was added to each sample in 1.5 mL microcentrifuge polypropylene vial. The vials were vortexed shortly to check proper sealing, and then shaken vigorously in

the ThermoMixer at 2000 rpm at room temperature for 2 minutes. For proper phase separation, the centrifugation was performed at 19,000× *g* for 2 minutes. After the centrifugation, the upper ethyl acetate phase was discarded by 1 mL pipette. The extraction was repeated several times based on the experimental design. After the last centrifugation step, the upper ethyl acetate phase was discarded by 1 mL pipette and the rest of the upper phase was carefully removed by a pipette with a gel loading tip.

Final steps
The lower aqueous phase containing cleaned peptides was used for peptide concentration assay, and finally evaporated completely on the SpeedVac vacuum concentrator.

SP2 PEPTIDE UNIVERSAL CLEAN-UP PROTOCOL
Removal of **polyethylene glycols** up to 1% **or SDS** up to 5%

Materials
2 mL Low-Binding Microcentrifuge Tubes with V-shaped bottom (SSIbio, cat.no. 1310-10, or similar)
Sera-Mag SpeedBeads Carboxylate-Modified Particles, 50 µg/µL, hydrophilic (Cytiva, 45152105050250)
Sera-Mag SpeedBeads Carboxylate-Modified Particles, 50 µg/µL, hydrophobic (Cytiva, 65152105050250)
Acetonitrile (LC-MS grade)
MilliQ water
Pure Proteome Magnetic Stand (Merck, cat.no. LSKMAGS08)
ThermoMixer C with ThermoTop (Eppendorf)
Savant SpeedVac SPD130DLX Vacuum Concentrator (ThermoFisher Scientific)

Dilution solution: Milli-Q water
Binding solution: 95% ACN
Washing solution: 100% ACN
Elution solution: 2% ACN in water

Beads wash

- remove beads in original bottles from the fridge, let stand for 10 min at RT and shake for 10 min at RT (hand mix)
- take 200 µL (10 mg) of **each** original bead stock and combine into a single 2 mL transparent microtube (V-shaped bottom) making 20 mg solids per tube in total
- place the tube on a magnetic rack for 1 minute to pellet the beads, and remove the supernatant on the rack
- off magnetic rack, reconstitute the beads in 2 mL of water and mix up and down with pipette
- on magnetic rack for 1 min pellet the beads, remove the supernatant on the rack by pipette

- off the rack reconstitute the beads in 400 µL water, resuspend the beads at concentration of **50 µg/µL**, and store at 4°C. Sufficient for about 60 peptide samples. *Prepared particles can be stored at 4°C for 6 months. Do not freeze the particles.*

Peptide Clean-Up

- estimate the **peptide concentration** (tryptophan assay)
- put **15 µL peptide solution (contains 10 ng up to 10 µg** of peptides) into 2 mL microtube (V-shaped bottom)
- add **6 µL of washed beads**, results in total volume of **21 µL**
- mix peptide sample and particle suspension, keep the liquid at the microtube bottom

Binding

- add **399 µL 100% acetonitrile** to obtain a final concentration of **95% acetonitrile**.
- mix by pipetting up and down to ensure particles are well dispersed, **when all the samples are done** allow the mixture to **settle for 5 min**
- place the microtube on the magnetic rack for 2 min
- remove the supernatant

Washing

- remove the microtube from the magnetic rack and add **500 µL** of **100% acetonitrile**.
- mix and **when all the samples are done** let **settle for 2 min**
- leave on the magnetic stand for 2 min
- remove the supernatant
- wash once more with **500 µL** of **100% ACN**. Remove the supernatant on the magnetic rack.

Elution

- **remove** the microtube from the magnetic rack
- add **54 µL** of **2% ACN/water** (e.g., 1.96 mL water + 40 µL ACN)
- **vortex** microtube for **30 s** to disperse beads (or vortex the whole magnetic rack pressing the one side of the stand against the Vortex **broad black rubber circle**)
- **settle** on the bench for **2 min**
- **briefly centrifuge** the samples for **3 s** using a benchtop mini centrifuge (no drops on the microtube wall)
- allow to **settle** the beads for **2 min**
- put on the magnetic rack for another **2 min**
- transfer **cleaned peptides** to a **fresh 0.5 mL microtube**
- **remove** the microtube from the magnetic rack
- add again **54 µL** of **2% ACN/water (the second elution)**
- **vortex** microtube for **30 s** to disperse beads (or vortex the whole magnetic rack pressing the
- one side of the stand against the Vortex black rubber circle)
- **settle** on the bench for **2 min**
- **briefly centrifuge** the samples for **3 s** using a benchtop mini centrifuge (no drops on the microtube wall)
- allow to **settle** the beads for **2 min**
- leave on the magnetic rack for another **2 min**

- add **cleaned peptides** to the **first eluate into 0.5 mL microtube** (total volume of cleaned peptides will be approx. 108 µL in **2% ACN**)
- **spin** 10 min at **20,000**× ***g***
- transfer the supernatant to a **new 0.5 mL** vial for MS analysis
- take aliquot for QC
- **dry to dryness** in the SpeedVac for acidic extraction/MS

Table S1. Average numbers of the peptides identified in 3 replicates (* in case of 1% PEG 2 replicates only) for 250 ng and 10 µg peptide inputs, and their standard deviations (SD). The numbers of peptides identified in individual conditions were compared relatively to control (no contamination, no clean-up, 100%). Experiments description: EE3 or EE12 stands for 3 or 12 EE iterations, respectively; control means samples with no contamination and no EE or SP2 processing; no SDS/PEG means samples subjected to clean-up by either EE or SP2 processing with no added contamination; 0.01, 0.1%, 1% or 5% represent samples with the given level of SDS or PEG contamination (v/v).

		PEPTIDE INPUT			
		250 ng		10 µg	
Experiment		Avg. No. of peptides ± SD	%	Avg. No. of peptides ± SD	%
**EE3** (SDS)	control	16,569 ± 542	100.0	41,331 ± 163	100.0
	no SDS	14,165 ± 567	85.5	34,369 ± 272	83.2
	0.1%	15,469 ± 307	93.4	34,543 ± 265	83.6
	1%	9491 ± 2260	57.3	31,693 ± 1156	76.7
**EE12** (SDS)	control	19,309 ± 1129	100.0	41,331 ± 163	100.0
	no SDS	13,406 ± 1185	69.4	32,608 ± 513	78.9
	0.1%	14,284 ± 1057	74.0	33,144 ± 345	80.2
	1%	13,857 ± 1108	71.8	33,063 ± 330	80.0
	5%	7228 ± 816	37.4	31,998 ± 593	77.4
**SP2** (SDS)	control	25,146 ± 410	100.0	41,331 ± 163	100.0
	no SDS	19,784 ± 335	78.7	40,453 ± 226	97.9
	0.1%	22,531 ± 1046	89.6	40,843 ± 300	98.8
	1%	22,446 ± 743	89.3	40,383 ± 454	97.7
	5%	23,029 ± 447	91.6	39,990 ± 341	96.8
**SP2** (PEG)	control	24,544 ± 193	100.0	41,331 ± 163	100.0
	no PEG	20,709 ± 256	84.4	40,453 ± 226	97.9
	0.01%	25,582 ± 258	104.2	40,847 ± 170	98.8
	0.1%	25,637 ± 570	104.5	40,687 ± 296	98.4
	1%	23,817 ± 384*	97.0	40,315 ± 295	97.5
**SP2** (PEG + SDS)	control	26,210 ± 557	100.0	41,331 ± 163	100.0
	no SDS + no PEG	22,068 ± 920	84.2	40,453 ± 226	97.9
	5% SDS + 1% PEG	25,453 ± 322	97.1	39,683 ± 525	96.0

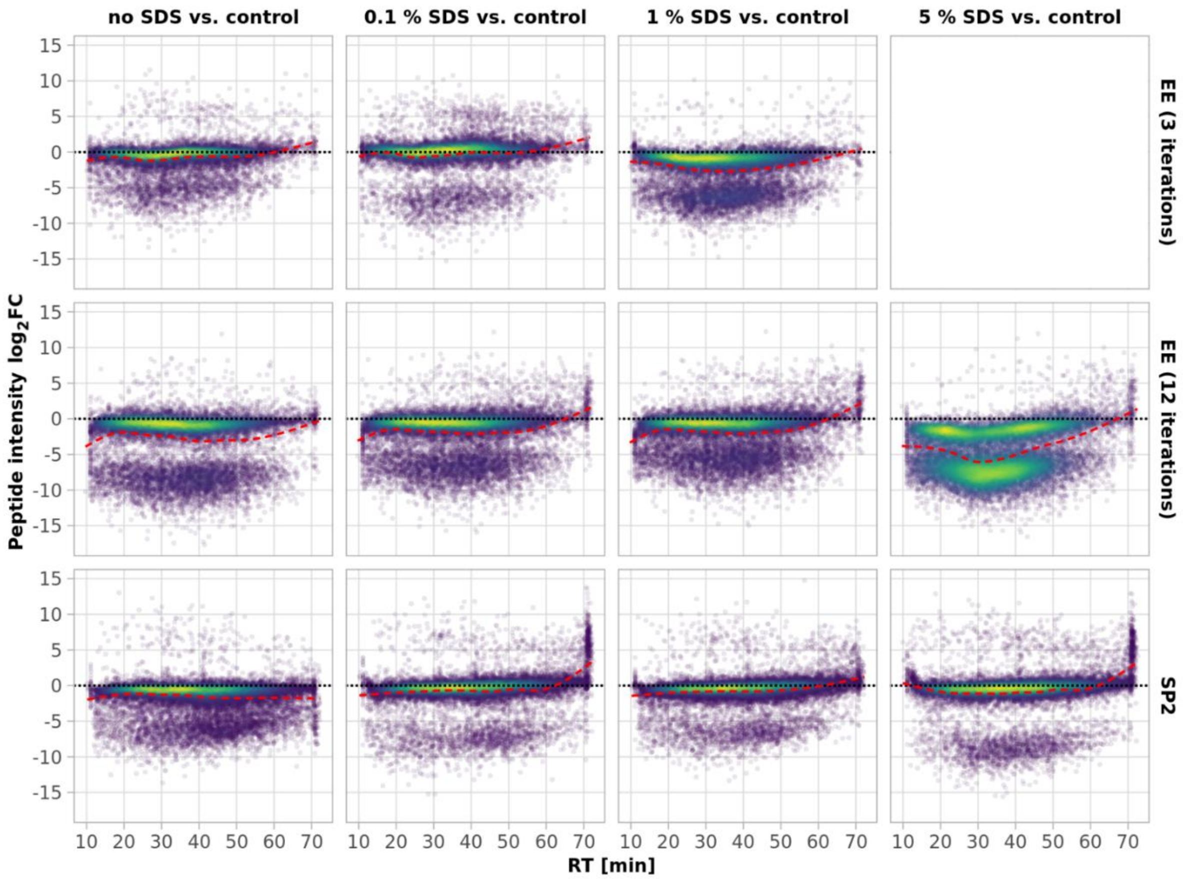

Figure S1. SDS clean-up of 250 ng peptide input by ethyl acetate extraction (3 and 12 iterations) and by SP2 - scatter plots of peptide intensities fold changes versus retention time (fold changes are $\log_2$ transformed, calculated from the comparison of given condition vs. control, i.e. uncontaminated and unprocessed sample). Only peptides quantified in at least 2 replicates out of 3 in either control or a sample were used for the fold change calculation. Missing values were imputed by a local minimum (the imputation led to formation of a seperated cloud/cluster of peptides with decreased $\log_2$FCs, e.g., after EE with 12 iterations of a sample contaminated by a 5% SDS). The black dotted lines denote no change (i.e., fold change = 1, $\log_2$FC = 0). The red dashed line represents the smoothed trend lines (method GAM) shown in the Figure 2A, B and C.

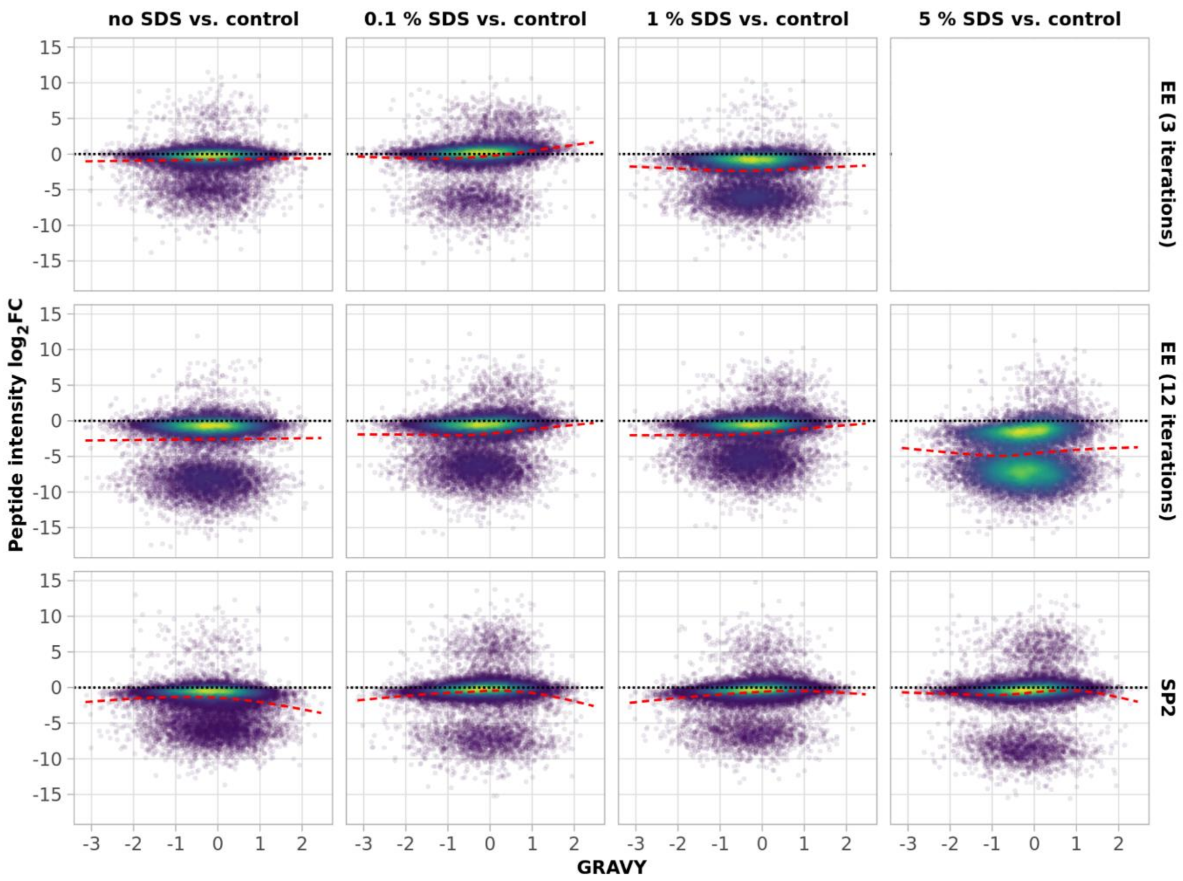

Figure S2. SDS clean-up of 250 ng peptide input by ethyl acetate extraction (3 and 12 iterations) and by SP2 - scatter plots of peptide intensities fold changes versus GRAVY index (fold changes are log_2 transformed, see the Figure S1 for more information about the fold changes calculation). The black dotted lines denote no change (i.e., fold change = 1, $log_2FC = 0$). The red dashed lines represent the smoothed trend lines (method GAM).

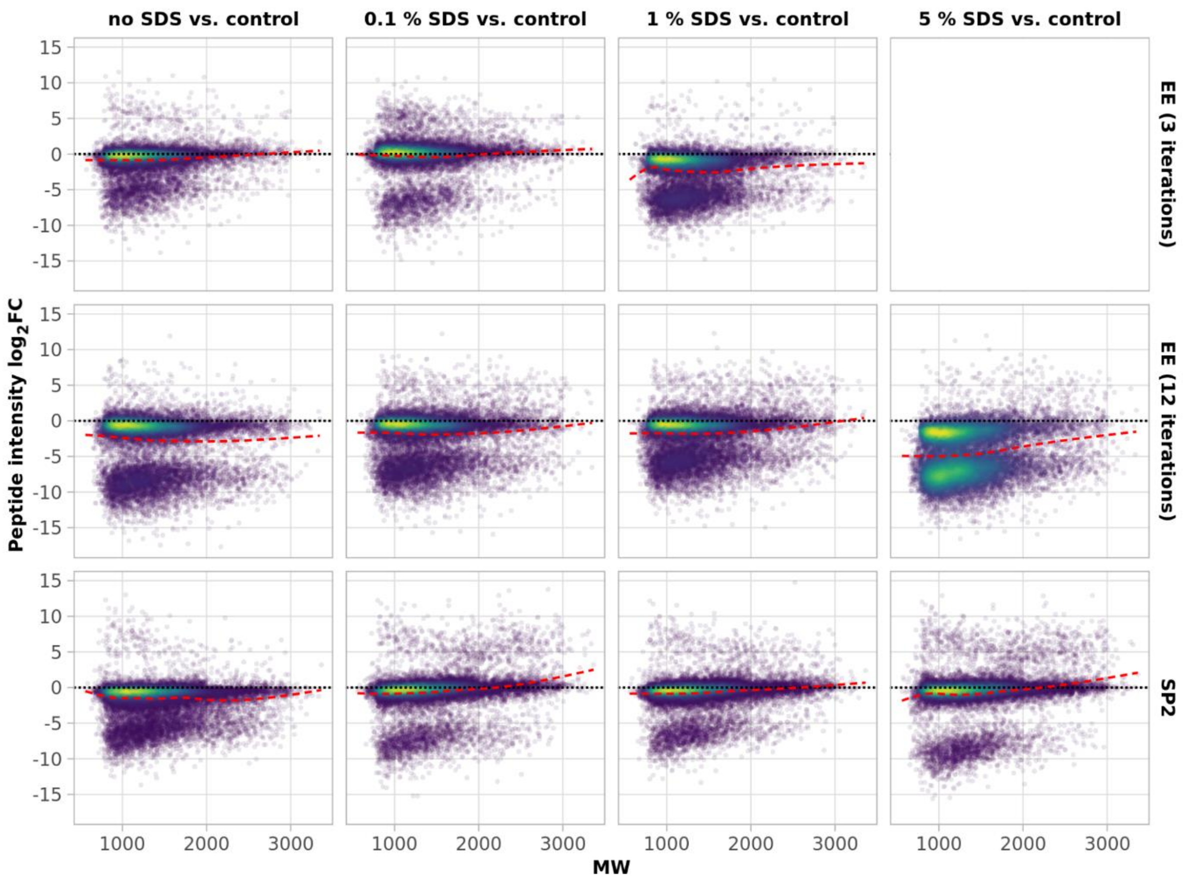

Figure S3. SDS clean-up of 250 ng peptide input by ethyl acetate extraction (3 and 12 iterations) and by SP2 - scatter plots of peptide intensities fold changes versus MW (fold changes are log_2 transformed, see the Figure S1 for more information about the fold changes calculation). The black dotted lines denote no change (i.e., fold change = 1, $log_2FC = 0$). The red dashed lines represent the smoothed trend lines (method GAM).

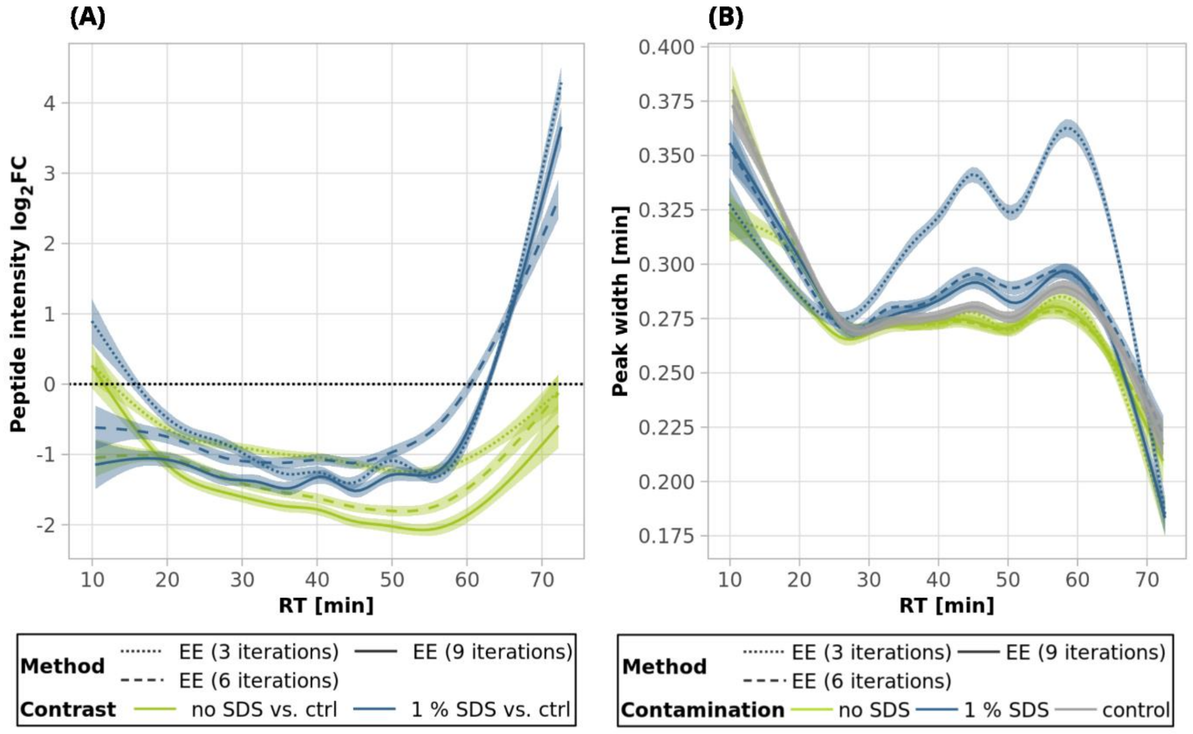

Figure S4. Optimization of ethyl acetate extraction iterations of 250ng peptide input contaminated by 1% SDS. Smoothed trend lines (method GAM) of peptide intensities fold changes (log_2 transformed, see the Figure S1 for more information about the fold changes calculation), calculated from the comparison of given condition vs. control (A) and peaks widths (B). The confidence intervals (level 0.95) are represented by the shaded area alongside the curves. The black dotted lines denote no change (i.e., fold change = 1, $log_2FC = 0$).

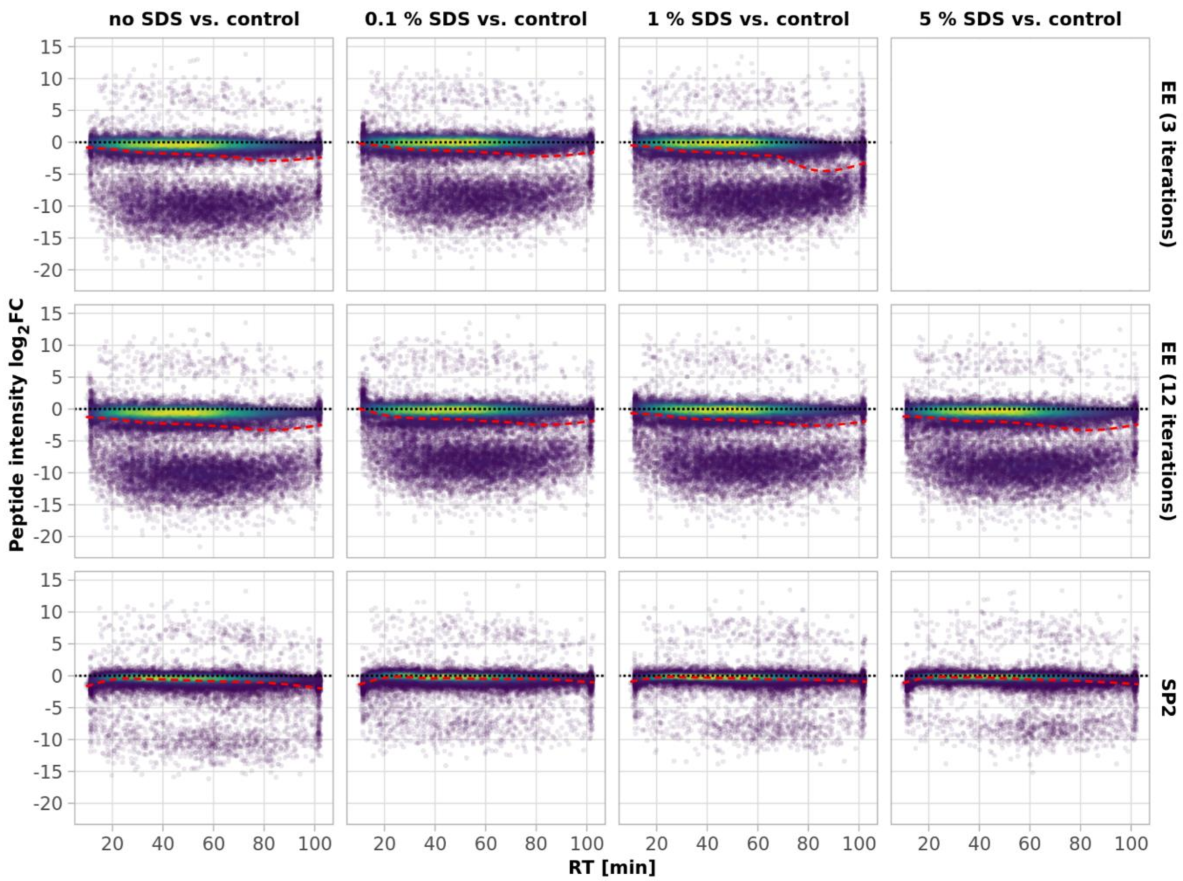

Figure S5. SDS clean-up of 10 µg peptide input by ethyl acetate extraction (3 and 12 iterations) and by SP2 - scatter plots of peptide intensities fold changes versus retention time (fold changes are log_2 transformed, calculated from the comparison of given condition vs. control). The red dashed line represents the smoothed trend lines (method GAM) shown in the Figure 3A, B and C. See Figure S1 for the detailed description.

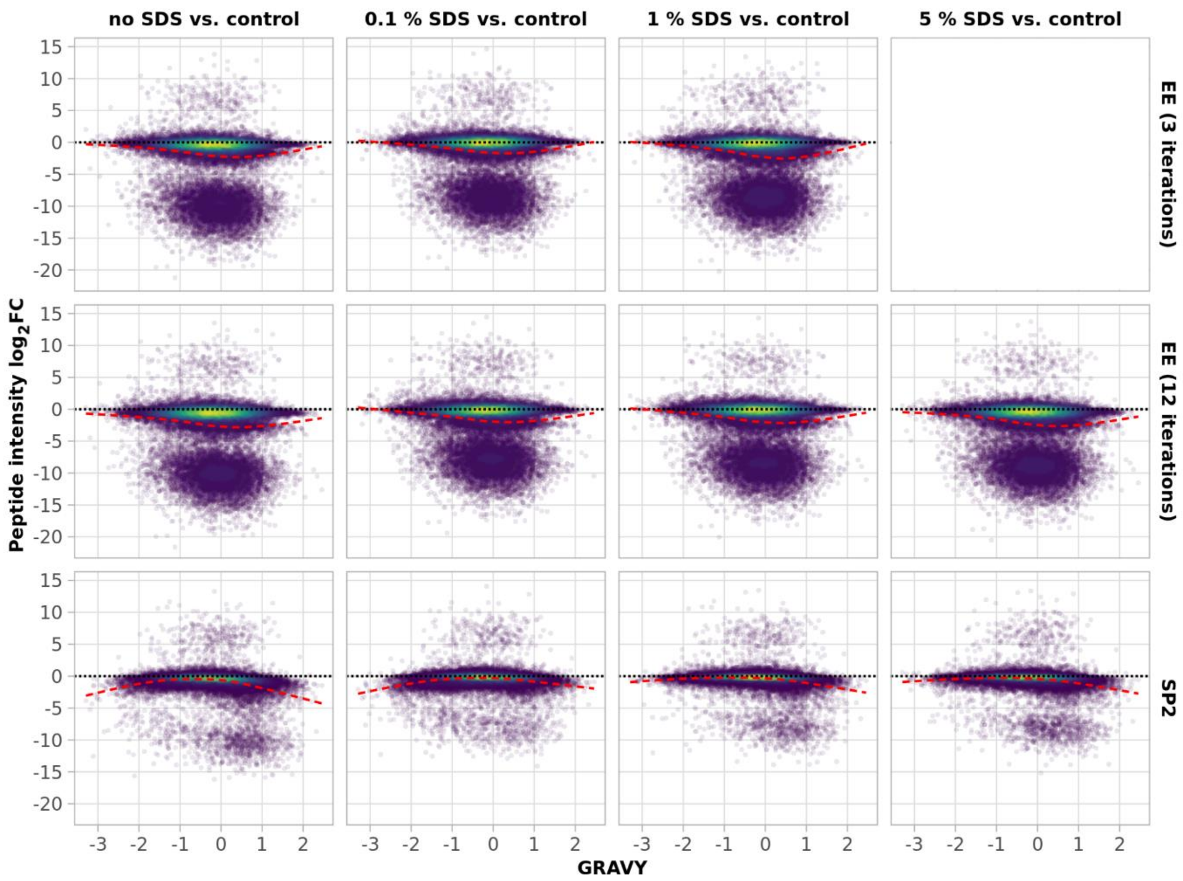

Figure S6. SDS clean-up of 10 µg peptide input by ethyl acetate extraction (3 and 12 iterations) and by SP2 - scatter plots of peptide intensities fold changes versus GRAVY index (fold changes are log_2 transformed, see the Figure S1 for more information about the fold changes calculation). The black dotted lines denote no change (i.e., fold change = 1, log_2FC = 0). The red dashed lines represent the smoothed trend lines (method GAM).

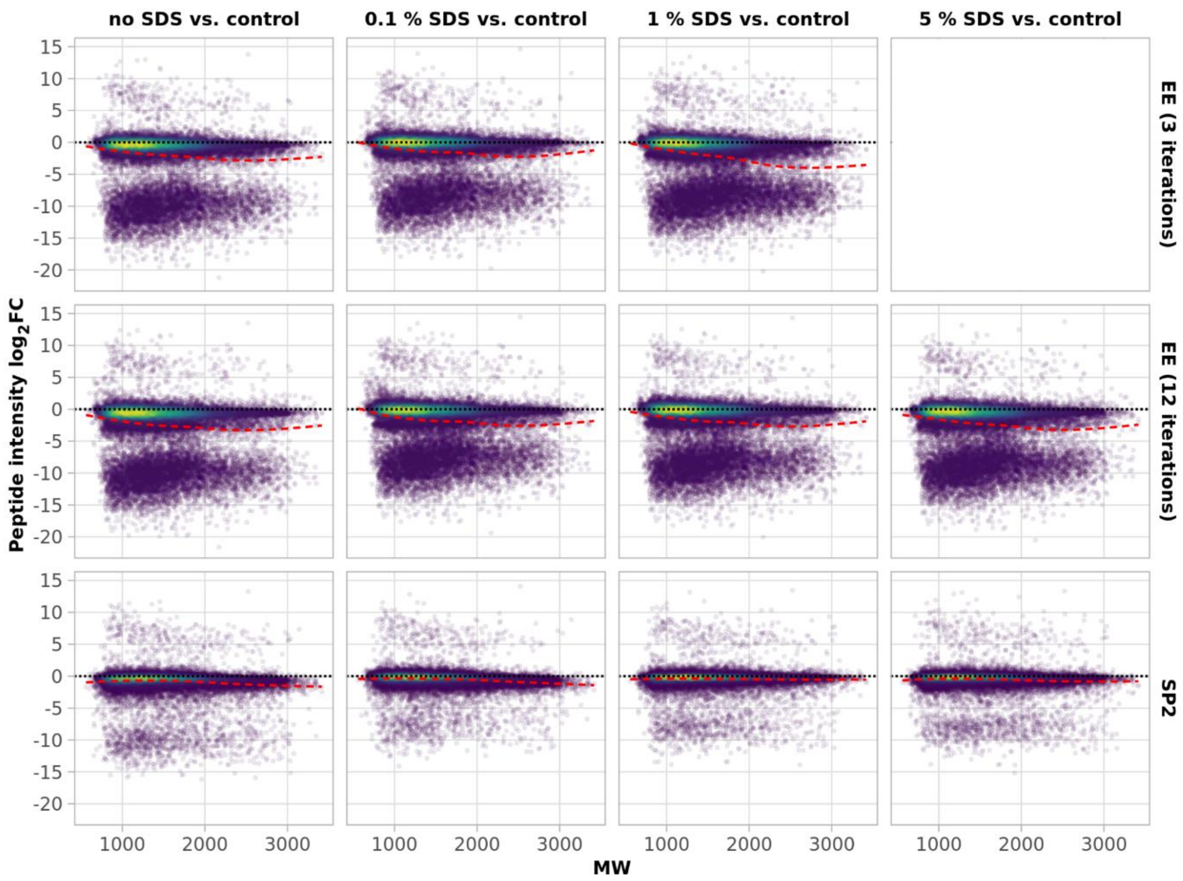

Figure S7. SDS clean-up of 10 µg peptide input by ethyl acetate extraction (3 and 12 iterations) and by SP2 - scatter plots of peptide intensities fold changes versus MW (fold changes are $\log_2$ transformed, see the Figure S1 for more information about the fold changes calculation). The black dotted lines denote no change (i.e., fold change = 1, $\log_2 FC = 0$). The red dashed lines represent the smoothed trend lines (method GAM).

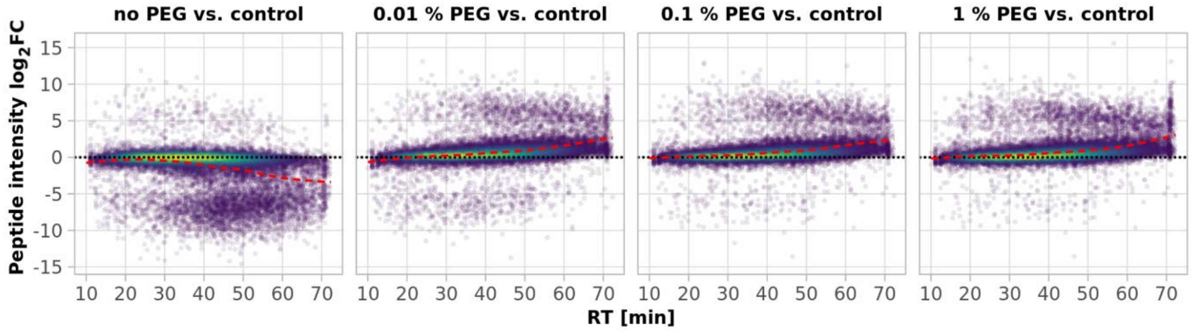

Figure S8. PEG clean-up of 250 ng peptide input by SP2 - scatter plots of peptide intensities fold changes versus retention time (fold changes are log_2 transformed, calculated from the comparison of given condition vs. control, i.e. uncontaminated and unprocessed sample). Only peptides quantified in at least 2 replicates out of 3 in either control or a sample were used for the fold change calculation (with the exception of 1% PEG, where only two replicates were available, in this case only peptides quantified in either at least 1 replicate of 1% PEG or 2 replicates of control passed the filter). Missing values were imputed by a local minimum. The black dotted lines denote no change (i.e., fold change = 1, $log_2FC = 0$). The red dashed line represents the smoothed trend lines (method GAM) shown in the Figure 5A.

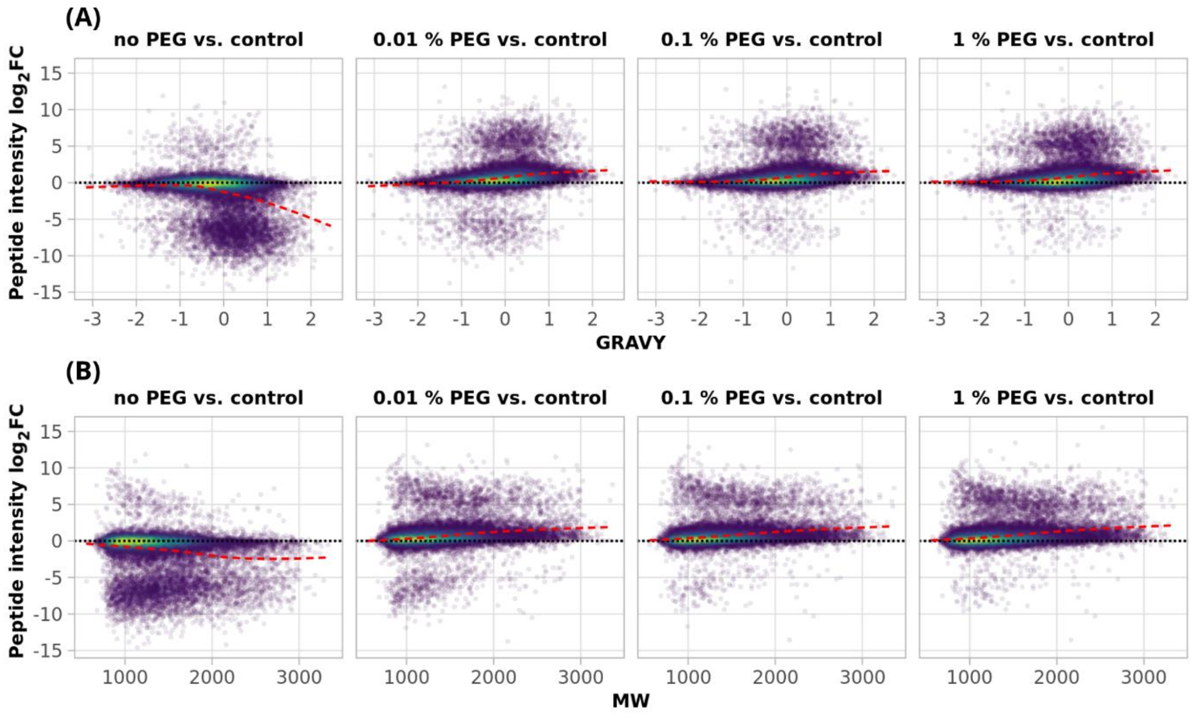

Figure S9. PEG clean-up of 250 ng peptide input by SP2 - scatter plots of peptide intensities fold changes versus GRAVY index (A) and MW (B; fold changes are log_2 transformed, see the Figure S8 for more information about the fold changes calculation). The black dotted lines denote no change (i.e., fold change = 1, $log_2FC = 0$). The red dashed lines represent the smoothed trend lines (method GAM).

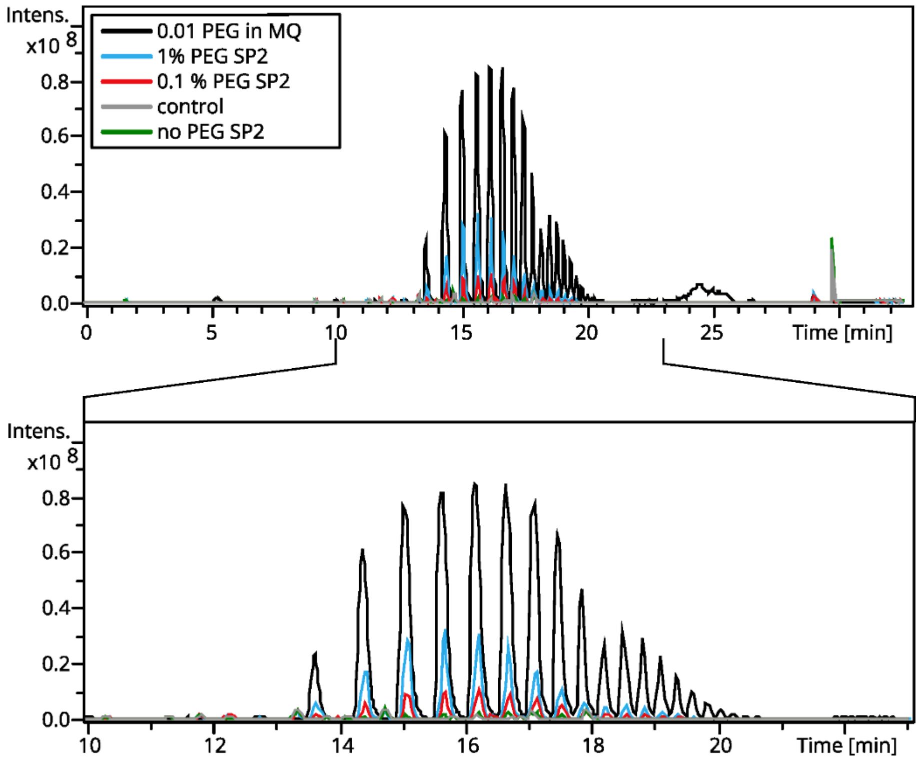

Figure S10. Quality control LC-MS base peak chromatograms of 250ng peptide input samples contaminated by 0.1 (red) or 1% (blue) PEG and without the PEG contamination (green) after SP2 cleaning step, and control (uncontaminated and unprocessed sample, grey). The black chromatogram represents 0.01% mixture of the PEGs used for artificial contamination of peptides (in water, see the section 4.5 Contamination of Peptides in Materials and Methods) for comparison.

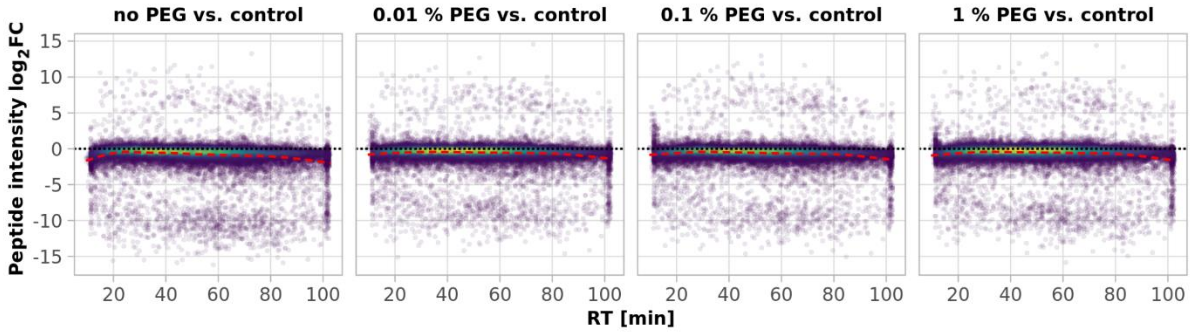

Figure S11. PEG clean-up of 10 µg peptide input by SP2 - scatter plots of peptide intensities fold changes versus retention time (fold changes are $\log_2$ transformed, see the Figure S1 for more information about the fold changes calculation). The black dotted lines denote no change (i.e., fold change = 1, $\log_2 FC = 0$). The red dashed line represents the smoothed trend lines (method GAM) shown in the Figure 5C.

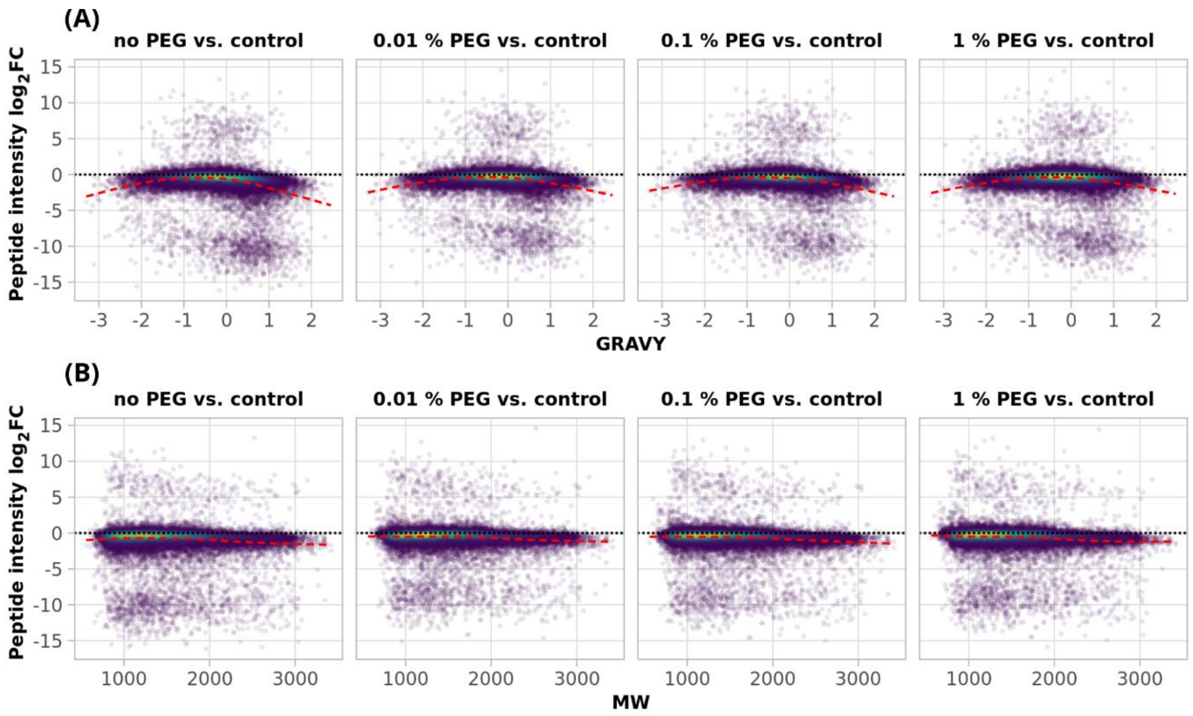

Figure S12. PEG clean-up of 10 µg peptide input by SP2 - scatter plots of peptide intensities fold changes versus GRAVY index (A) and MW (B; fold changes are $\log_2$ transformed, see the Figure S1 for more information about the fold changes calculation). The black dotted lines denote no change (i.e., fold change = 1, $\log_2FC = 0$). The red dashed lines represent the smoothed trend lines (method GAM).

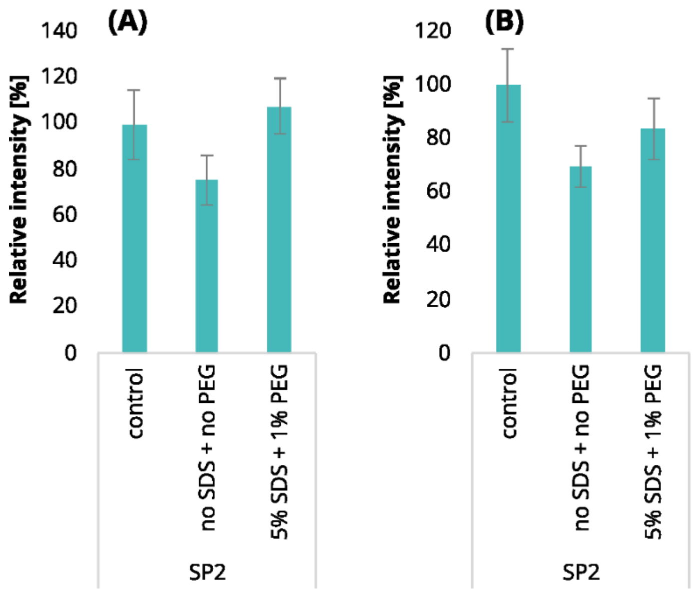

Figure S13. SP2 clean-up of simultaneous contamination by 5% SDS and 1% PEG. Mean intensity sums of quantified peptides for 250 ng (A) and 10 µg (B) peptide input. The mean sum intensities were compared to control (100%). Standard deviations are displayed as error bars.

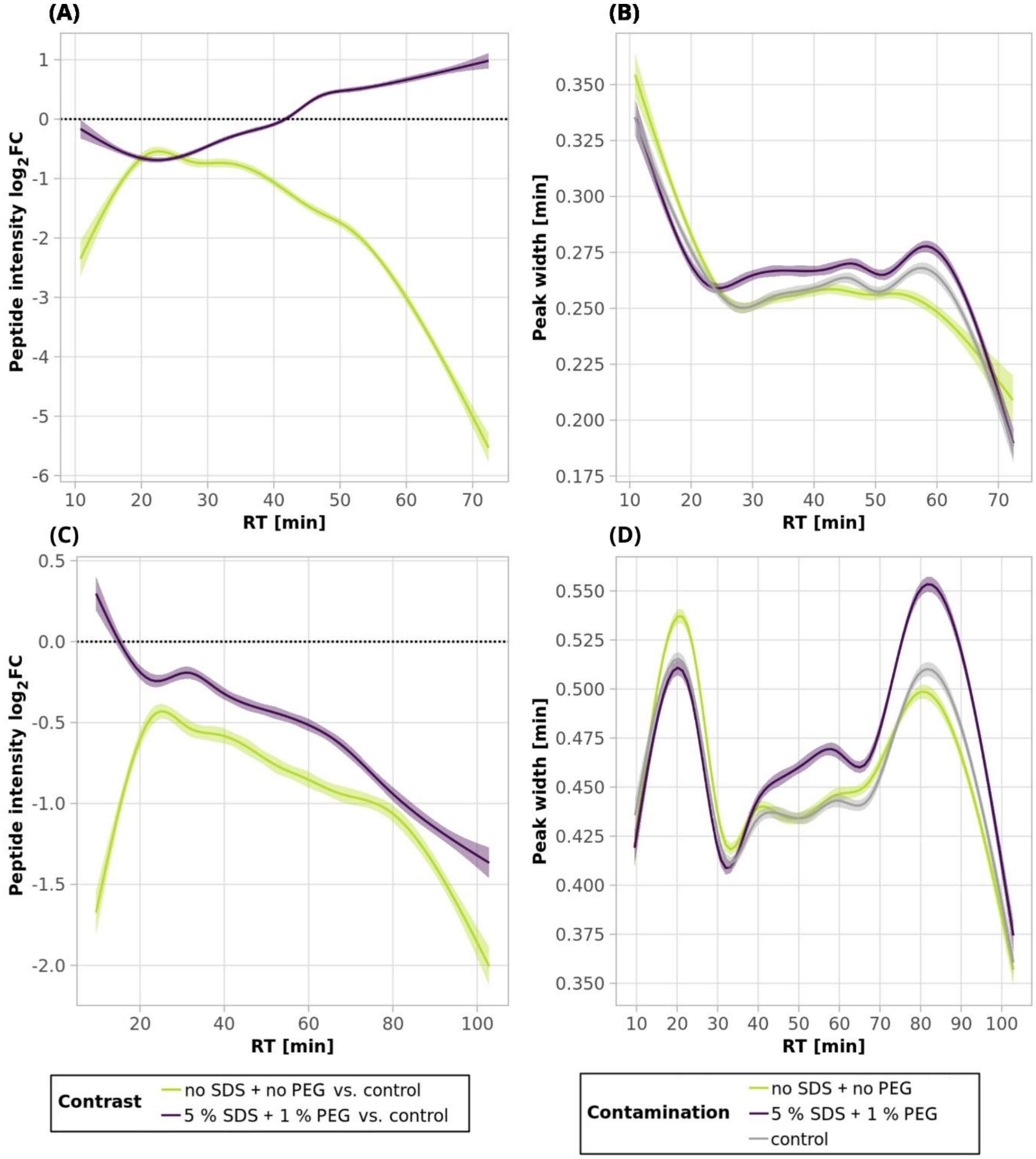

Figure S14. SP2 clean up of simultaneous contamination by 5% SDS and 1% PEG of 250 ng (A–B) and 10 µg (C–D) peptide inputs - smoothed trend lines (method GAM) of peptides intensities fold changes (log_2 transformed, calculated from the comparison of given condition vs. control; A, C), and **peaks widths** (B, D) for SP2 clean up of 250 ng (A–B) and 10 µg (B–D) peptide inputs contaminated simultaneously by 5% SDS and 1% PEG. Only peptides quantified in at least 2 replicates out of 3 in either control or a sample were used for the fold change calculation. Missing values were imputed by a local minimum. The confidence intervals (level 0.95) are represented by the shaded area alongside the lines. The black dotted lines denote no change (i.e., fold change = 1, log_2FC = 0).

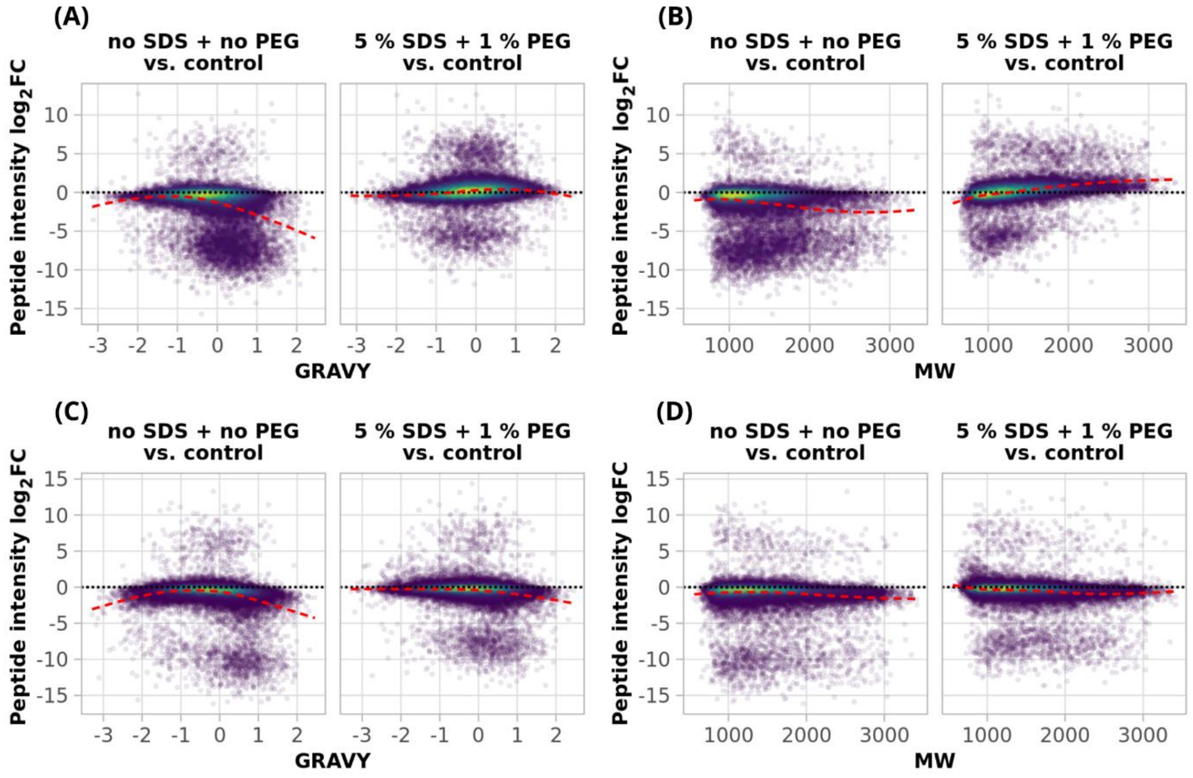

Figure S15. SP2 clean up of simultaneous contamination by 5% SDS and 1% PEG of 250 ng (A–B) and 10 µg (C–D) peptide inputs - scatter plots of peptide intensities fold changes versus GRAVY index (A, C) and **MW** (B, D**;** fold changes are $\log_2$ transformed, see the Figure S1 for more information about the fold changes calculation). The black dotted lines denote no change (i.e., fold change = 1, $\log_2 FC = 0$). The red dashed lines represent the smoothed trend lines (method GAM).

Table S2. Average numbers of the peptides identified in 3 replicates (* in case of control it was 2 replicates) for 10 ng peptide input and their standard deviations (SD). The numbers of peptides identified in individual conditions were compared relatively to control (no contamination, no clean-up, 100%). See Table 1 for the experiments' description.

Experiment		Avg. No. of peptides ± SD	%
**SP2** (SDS)	control*	3996 ± 478	100.0
	no SDS	3720 ± 116	93.1
	0.1%	3855 ± 65	96.5
	1%	1056 ± 448	26.4
**SP2** (PEG)	control*	3996 ± 478	100.0
	no PEG	3720 ± 116	93.1
	0.01%	4313 ± 406	107.9
	0.1%	4490 ± 57	112.4
